# Problematic Mobile Phone and Smartphone Use Scales: A Systematic Review

**DOI:** 10.3389/fpsyg.2020.00672

**Published:** 2020-05-05

**Authors:** Bethany Harris, Timothy Regan, Jordan Schueler, Sherecce A. Fields

**Affiliations:** ^1^Department of Psychological & Brain Sciences, Texas A&M University, College Station, TX, United States; ^2^Department of Psychology, Texas A&M University, College Station, TX, United States

**Keywords:** mobile phone, smartphone, problematic use, addiction, assessment

## Abstract

The popularity of smartphones is undeniable in nearly all facets of society. Despite the many benefits attributed to the technology, concern has grown over the potential for excessive smartphone use to become problematic in nature. Due to the growing concerns surrounding the recognized and unrecognized implications of smartphone use, great efforts have been made through research to evaluate, label and identify problematic smartphone use mostly through the development and administration of scales assessing the behavior. This study examines 78 existing validated scales that have been developed over the past 13 years to measure, identify or characterize excessive or problematic smartphone use by evaluating their theoretical foundations and their psychometric properties. Our review determined that, despite an abundance of self-report scales examining the construct, many published scales lack sufficient internal consistency and test-retest reliability. Additionally, there is a lack of research supporting the theoretical foundation of many of the scales evaluated. Future research is needed to better characterize problematic smartphone use so that assessment tools can be more efficiently developed to evaluate the behavior in order to avoid the excessive publication of seemingly redundant assessment tools.

## Introduction

Smartphone ownership has become increasingly more prevalent over the past decade since Apple’s first iPhone smartphone device was launched in 2007 (Apple Inc, 2007). In 2018, the Consumer Technology Association (CTA) revealed that smartphones were owned in 87% of United States homes and predicted that smartphone ownership could reach household TV ownership rates (96%) within 5 years (Twice Staff, 2018). However, in the fields of psychology and cognition, it is not the mere ownership of the technological devices that is causing increased concern. It is, instead, the potential for dysfunction associated with smartphone use that is leading researchers to stress the importance of investigating the behavior. Therefore, the purpose of this paper is 3-fold. First, we review literature examining psychological and behavioral dysfunctions related to smartphone use as well as probe the potential role problematic smartphone usage may occupy within the realm of addiction research. Second, we present an exhaustive review of assessment scales that measure problematic smartphone or mobile phone use including an overview of reliability (i.e., internal consistency and test-retest reliability) and criterion-related validity by each scale. Third, we will provide specific recommendations for moving the field forward including furthering research in order to standardize conceptualization of the behavior.

### Associated Dysfunction

It is important to note that a standard cut-off point to determine when smartphone use becomes problematic has yet to be established. Due to insufficient research investigating problematic smartphone use in order to effectively and consistently characterize it, it is currently unclear whether “problematic use” ought to be defined by use quantity, patterns of use, or by the negative consequences of the use. Billieux (2012) conducted a frequently cited literature review of dysfunctional mobile phone use and defined the problematic use of mobile phones as “an inability to regulate one’s use of the mobile phone, which eventually involves negative consequences in daily life” (pg. 1). Numerous research studies indicating that smartphone use is related to various facets of dysfunction support Billieux’s (2012) conceptualization of problematic use being contingent upon negative consequences associated with the use. Evidence has accumulated showing strong links between smartphone use and social, interpersonal, mental health, cognition and academic dysfunction, suggesting that smartphone use can result in significant negative consequences for some individuals (see review, Billieux, 2012).

For example, although smartphones provide unique opportunities for social interaction, Scott et al. (2016) found that problematic attachment to technology such as smartphone devices was associated with lowered social skills, emotional intelligence and empathy, as well as increased conflict with others. Additionally, Laramie (2007) identified social anxiety and loneliness as being associated with heavy use of and reliance upon mobile phones, suggesting smartphone overuse may result in interpersonal dysfunction. Relatedly, self-reported subjective smartphone addiction has been shown to be negatively correlated with psychological well-being (Kumcagiz and Gündüz, 2016). Several studies have revealed evidence that low self-esteem (Bianchi and Phillips, 2005; Hong et al., 2012). and depression and anxiety (De-Sola et al., 2017b; Elhai et al., 2017; Matar Boumosleh and Jaalouk, 2017) are associated with problematic smartphone use, especially in populations of adolescents and young adults. The results of these studies present rationale for a justified concern surrounding potential negative psychological consequences of smartphone overuse.

Similarly, concern has grown over the potential negative impacts smartphone use might have on users’ behavior and cognitive abilities. Research has shown that problematic smartphone use is related to impulsivity (Contractor et al., 2017; De-Sola et al., 2017b; Hadar et al., 2017), impaired attention (Roberts et al., 2015; Hadar et al., 2017), and compromised inhibitory control (Chen et al., 2016). These associated cognitive deficits have spurred researchers to investigate the potential for dysfunction in academic performance, as well. Smartphone use has been shown to negatively correlate with academic progress and success (Alosaimi et al., 2016; Hawi and Samaha, 2016; Samaha and Hawi, 2016). Findings from these studies suggest the cognitive dysfunction associated with smartphone overuse may result in real-world consequences for some individuals.

To reiterate, despite research efforts characterizing associated dysfunction, a standardized conceptualization of problematic smartphone use has yet to be established in the field. However, the previously described areas of dysfunction (e.g., social, interpersonal, mental health, cognition, and academia) found to be associated with smartphone use support Billieux’s (2012) conceptualization of problematic smartphone use being contingent upon negative consequences associated with the use. As such, many assessment tools for problematic use tap into these types of negative life consequences as they are likely to identify individuals for which excessive smartphone use is especially harmful.

### Is Smartphone Addiction a Real Concept?

The American Psychiatric Association (APA) broadly defines addiction as “a complex condition, a brain disease that is manifested by compulsive substance use despite harmful consequences” (pg. 1; American Psychiatric Association, 2017). In this definition, the use of substances is a requirement of the condition in that, to be “addicted,” one must have a substance to which to be addicted. But, what about behavioral addictions? Both the Diagnostic and Statistical Manual of Mental Disorders (DSM-5; American Psychiatric Association, 2013) and the International Classification of Diseases (ICD-11; World Health Organization [WHO], 2018) have grouped behavioral addictions within their respective substance dependence categories. Re-categorization of addictions was seen in the DSM-5 resulting in gambling disorder being recognized as a non-substance-related addictive disorder (American Psychiatric Association, 2013). Additionally, Internet Gaming Disorder (IGD) is included in the DSM-5 as a condition for further study (American Psychiatric Association, 2013). Finally, both gambling disorder and gaming disorder are grouped together in the ICD-11 (World Health Organization [WHO], 2018), suggesting behavioral addictions share some common ground with substance use disorders (SUD).

Despite this conceptual similarity, Billieux et al. (2015) argue that, while addictive behaviors like problematic smartphone use is associated with several types of associated dysfunction, research in this arena is inconsistent in documenting significant behavioral and neurobiological similarities and correlates with more widely recognized substance addictions. For example, there are many features of substance addiction that do not appear to be present when considering excessive smartphone use. Very little research has documented the presence of loss of control (i.e., trouble consciously limiting one’s smartphone use), tolerance (i.e., increasing smartphone use to achieve satisfaction), and withdrawal (i.e., negative symptoms that occur after smartphone use discontinuation; Billieux et al., 2015). Also, dependence symptoms such as tolerance and withdrawal, theoretically based in physiological adaptation to increasing amounts of a drug, are often absent in behavioral addictions. In their review for IGD, Kaptsis et al. (2016) did not find consistent answers to questions inquiring about withdrawal symptoms, such as effects on mood (i.e., feeling “irritable,” “dissatisfied,” or “moody” when unable to play a game) for IGD. Similarly, physiological and neurobiological adaptations to increasing amounts of smartphone use have yet to be documented, suggesting researchers may need to use other criterion to define problematic smartphone use. Some researchers have argued that “borrowing” such criteria from more recognized addictive behaviors, like substance abuse or problematic gambling, might not fit for certain problematic or excessive behaviors (Starcevic, 2016). Thus, although sharing common ground, problematic smartphone use may substantially differ from substance addiction in regards to loss of control, tolerance, and withdrawal.

Some other criteria for addiction map on better. Aforementioned associated life dysfunction is becoming increasingly documented, meaning the problematic use of smartphone devices has real-world negative consequences for some individuals. Compulsive use has been documented: Parasuraman et al. (2017) found that over 50% of participants would not quit using their smartphones even though their daily lifestyles were being negatively affected by their excessive use. This irresistible impulse to use one’s smartphone despite wanting to stop is reminiscent of individuals with SUDs, in which the drive to use drugs overrides other executive control processes. Six symptom criteria were even proposed to diagnose smartphone “addiction” and related functional impairment, which were based on guidelines for SUDs and IGD. Lin et al. (2016) dropped tolerance from their final criterion, due to low diagnostic accuracy. However, they included withdrawal, as subjects who used their smartphones excessively enough to be considered “addicted” displayed feelings of dysphoria, anxiety, and/or irritability after a period without their smartphones.

Dependency appears, to some extent, in excess smartphone users, although again this is based on subjective self-report. In a study conducted by Parasuraman et al. (2017) analyzing smartphone use behavior, almost 75% of smartphone users reported feeling dependent upon smartphone devices and 58% of users felt as though they were “unable to withstand” not having their smartphone with them. Additionally, over 70% of participants indicated that they use their smartphone longer than they intended. Similarly, results from a research study released by *The Sun* newspaper in March of 2013 indicated that one in ten college students say that they are “addicted” to their smartphones (Hope, 2013). Upon surveying 2,000 college students, 85% of the students endorsed the question about constantly checking their smartphones to figure out what time it is and 75% of the students responded that they sleep with their smartphones lying beside them. These data indicate, when used excessively, smartphones can become problematic and users report feeling as though they have an addiction to them.

Laws have even been enacted in many states to combat problematic use. Phone use while driving a vehicle has become a major concern and it has been shown that it is the anticipation of incoming calls, messages and notifications that directly correlates with greater in-vehicle phone use (O’Connor et al., 2013, 2017). Additionally, recently, the city of Honolulu, Hawaii has even gone so far as to enact a law making it illegal for pedestrians to use their phones when crossing a street or highway (Honolulu, Hawaii, Ordinance 17-39, Bill 6, 2017) due to the significant increase in pedestrian fatalities in the city partially attributed to smartphone distraction (Ellis, 2017). Thus, more and more individuals are using their smartphones in risky and physically hazardous situations. This is conceptually similar to some more recognized addiction criteria for SUDs in DSM-5.

### DSM-5 Criteria and Considerations

As reviewed, problematic smartphone use shares some conceptual similarities with more typically recognized addictions, including excessive use, failure of impulse control, feelings of dependency, use in risky and/or physically hazardous situations, and potential for negative affect when not using one’s smartphone. The term “addiction” is typically characterized by these criterion, but the question of whether “behavioral addictions” must contain all of these same criterion to be considered a true “addiction” is still under debate.

Starcevic (2013) suggested behavioral addictions are characterized by salient behaviors which promote craving and neglect of other life activity, loss of control, tolerance and withdrawal manifestations, and negative consequences from overuse. Gambling disorder, considered an impulse control disorder in the DSM-IV (American Psychiatric Association, 2000), is now characterized and grouped with SUDs in the most recent DSM-5 (American Psychiatric Association, 2013) in a new category of psychopathology entitled “Substance-Related and Addictive Disorders.” This transition was the result of a wide body of research demonstrating clinical, phenomenological, genetic, neurobiological, and other similarities between gambling disorder and SUDs (Potenza, 2014). While gambling disorder is currently the only representative member of the “Non-Substance-Related Disorders” subsection, this transition was an important shift for the recognition of “behavioral addictions” more broadly. Many researchers now advocate for the similar recognition of problematic smartphone use (e.g., Potenza et al., 2018).

Support for recognition of problematic smartphone use has also been motivated by the growing body of research literature on Internet addiction seen since the late 1990s. Kimberly Young is considered to be the “founder” of the concept of Internet addiction due to her publication of a case study in 1996 involving a 43-year-old female with no addiction or psychiatric history who abused the Internet causing significant impairment (Young, 1996). This led to her development and validation of the Young Internet Addiction Scale (Y-Scale; Young, 1998) assessing self-reported preoccupation with the Internet, need to use the Internet with increasing amounts of time, unsuccessful efforts to stop use, restlessness associated with decreased use, longer than intended use, associated life impairments, concealment of involvement, and use of the Internet to relieve a dysphoric mood. The scale’s items were derived from the DSM-IV’s criteria for Pathological Gambling (American Psychiatric Association, 1995) due to her conceptualization of the behavior as being similar to other impulse-control disorders. It seems likely that the development of this scale and the subsequent research that has been conducted on Internet addiction have greatly influenced the investigation of problematic smartphone use as a similar disorder.

In light of growing concerns surrounding the known and unknown implications of smartphone use as well as these recent changes in the conceptualization of non-substance-related addictions, great efforts have been made through research to identify, label and evaluate problematic smartphone use mostly through the development and administration of scales measuring and characterizing the behavior. Researchers within the past 13 years have set out to develop assessment tools based upon varying diagnostic criteria for officially recognized disorders and addictions such as SUDs and gambling disorder as well as unofficial criteria associated with Internet addiction. The aim of the present review is to examine existing validated scales that have been developed to measure, identify or characterize problematic smartphone use by evaluating their theoretical foundations and their psychometric properties.

## Methods

### Literature Collection

All studies (published between January 1994 and May 2019) validating standardized measures of varying forms of problematic smartphone use were identified by searching two databases (PsycINFO and MEDLINE Complete) through EBSCOhost. The date range was decided upon after conducting a preemptive literature search utilizing the search terms listed in Appendix A and concluding that the earliest study was published in 1994 (Clifford et al., 1994). For the EBSCOhost literature collection, language was limited to English. Further studies, including those in other languages, were identified by reviewing the bibliographies of relevant studies and reviews.

### Search Terms

Due to inconsistencies in the field regarding the conceptualization of the technology being used and the use of the technology, various terms were used in order to ensure that all relevant studies would be identified and reviewed. In addition to searching for studies identifying problematic use of smartphones, terms such as “smart phone,” “cellular phone,” “cell phone,” “mobile device,” and “mobile phone” were used. Additionally, because of the conceptualization of the problematic use has also been shown to be inconsistently described in research studies, terms such as “dependence,” “dependency,” “overuse,” “nomophobia,” “attachment,” and “compulsive” were used during the literature collection process. Finally, terms such as “questionnaire,” “scale,” “inventory,” measurement,” and “validation” were used to ensure all studies validating measurement scales were identified. The full search strategy is presented in Appendix A.

### Inclusion/Exclusion Criteria

Scales were selected for inclusion if: (a) their development and validation were investigated in the identified study, or (b) they were described in the methods section of a research study as being used to identify or evaluate the behavior. Scales were excluded from the systematic review when they were used to measure behavior not specific to smartphone or cellular phone problematic use.

### Data Extraction

Once a measurement scale was identified through the review of a study, a structured process was used to extract data on the scale (title, abbreviation, and the author(s) of original development/validation study), items (total number, format, and scale range), sample and norms (validation study participant count and descriptions), reliability (internal consistency and temporal stability), validity (content domains and criterion-related validity), and construct being measured. If a scale was mentioned in a research study as being used to measure the behavior, the study used to validate the scale and discuss its development was found in the study’s references and used to extract these data.

Format of the scale items was identified and described as either Likert scale (range of potential responses on a continuum) or dichotomous (yes or no response options). Internal consistency (the degree of the interrelatedness among the items; Mokkink et al., 2013) was assessed and the Cronbach’s alpha (α) value was recorded for each scale if provided in the validation study. Reported temporal stability, or test-retest reliability, measuring the stability of the responses to items over time was assessed and were recorded for each scale, as well. Content domains were often identified by using the factors listed by the author indicated through factor analysis of their scale’s items. The content domains often reflected similar criteria used to assess disorders or conditions claimed by the researchers to be similar in nature to the problematic behavior being assessed. The criterion-related validity (the degree to which the scores of the instrument are an adequate reflection of a “gold standard;” Mokkink et al., 2013) of each scale was identified by assessing the scales and criteria used by the researchers to validate their instruments. Finally, the purported construct being measured by each scale was typically identified by evaluating the title assigned to the scale by the researchers and their description of the purpose of developing the scale.

## Results

### Identification of Measurement Scales

**FIGURE 1:**
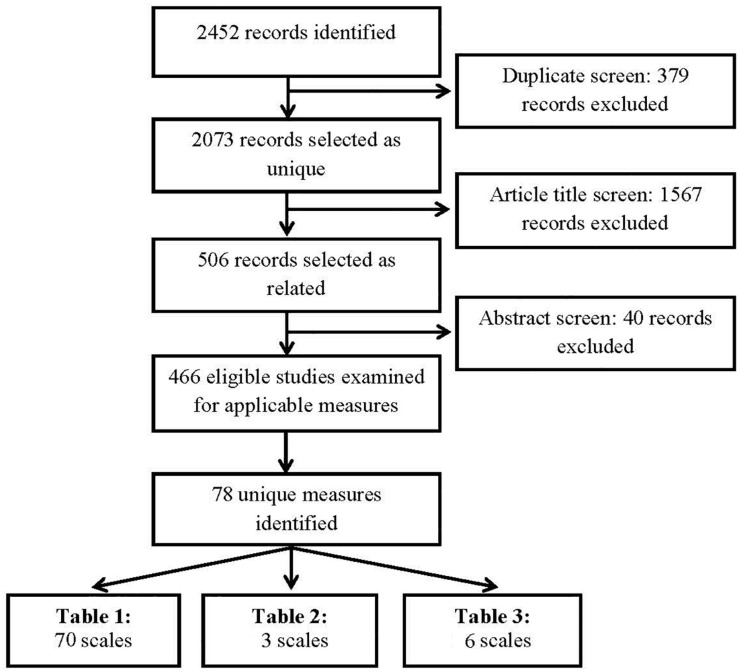
Study flow diagram showing review process on measures of problematic smartphone use.

The process for the identification and selection of the problematic smartphone use scales is displayed in the flow diagram (see Figure 1). The combined search strategy using PsycINFO and MEDLINE Complete databases and the search terms displayed in Appendix A yielded 2452 potentially relevant articles. From them, 379 duplicate articles were excluded leaving 2073 remaining articles identified as being unique. By screening the titles of the articles, 1567 articles were excluded leaving 506 articles identified as being potentially relevant. Next, through an abstract screening process, a single, broad exclusion criteria was utilized to evaluate article relevance and inclusion. Articles were eliminated if there was no mention of either development and/or validation or utilization of assessment tools examining use of smartphone or mobile phone devices in their abstract. For example, articles were eliminated if researchers utilized smartphone devices to administer assessments of unrelated constructs (e.g., depression, anxiety). This resulted in the removal of 40 articles. The remaining 466 articles were identified as being eligible studies requiring further examination in order to identify applicable measures. Finally, through an in-depth examination process, 78 total scales were identified as being unique and relevant. These scales are organized by purpose and can be found in Table 1 (Problematic Smartphone Use Measurement Scales; 70 scales), Table 2 (Smartphone Use Frequency Scales; 3 scales), and Table 3 (Smartphone Use Motivations and Attitudes Scales; 6 scales), with one scale (MTUAS; Rosen et al., 2013) appearing in both Tables 2, 3 due to overlapping constructs being measured.

**TABLE 1:** Problematic smartphone use measurement scales.

Title	Abbrev.	Author(s)	Items	Item format	Item scale	Content domains	Internal consistency (Cronbach’s α)	Temporal stability (Test-Retest)	Sample/Norms (Age: *M*, *SD*)	Purported construct	Criterion-Related validity
Cellular Phone Dependence Questionnaire	CPDQ	Toda et al. (2004)	20	Likert scale	0–3	Unknown	0.86	N/A	168 female university students (21.7 ± 2.6)	Dependence	Unreported
Mobile Phone Problem Use Scale	MPPUS	Bianchi and Phillips (2005)	27	Likert scale	1–10	Tolerance; Escape from other problems; Withdrawal; Craving; Negative life consequences	0.93	N/A	195 adult mobile-phone users (36.1 ± 12.4)	Problematic use	MMPI-2 Addiction Potential Scale (APS; Weed et al., 1992)
Self-Perception of Text-Message Dependency Scale	STDS	Igarashi et al. (2005)	15	Likert scale	1–5	Perception of emotional reaction; Excessive use; Relationship maintenance	Unreported	N/A	248 Japanese undergraduate students (Unreported)	Dependence	Unreported
Cell Phone Overuse Scale	COS	Jenaro et al. (2007)	23	Likert scale	1–6	Preoccupation; Tolerance; Lack of control; Withdrawal; Escape; Deception; Life dysfunction	0.87	N/A	337 Spanish college students (21.6 ± 2.5)	Excessive use	DSM-IV criteria for pathological gambling
SMS Problem Use Diagnostic Questionnaire	SMS-PUDQ	Rutland et al. (2007)	8	Dichotomous items	Yes/No	Relapse; Withdrawal; Interpersonal conflict; Mood modification; Salience: Preoccupation; Tolerance; Salience: Compulsivity	0.84 and 0.87	N/A	78 United States college students (20.7 ± unreported)	Compulsive use of SMS	Internet addiction
Problematic Mobile Phone Use Questionnaire	PMPUQ	Billieux et al. (2008)	30	Likert scale (plus 1 dichotomous)	1–4	Prohibited use; Dangerous use; Dependence; Financial problems	0.65–0.85	N/A	339 French-speaking young adults (25.8 ± 4.0)	Problematic use	Existing measurement scales for problematic phone use
Instant Messaging Technology Addiction	IMAT	Ehrenberg et al. (2008)	3	Likert scale	1–7	Salience; Loss of control; Withdrawal	0.69	N/A	200 undergraduate students (19.1 ± 1.8)	Instant Messaging addiction	Unreported
Excessive Cellular Phone Use Survey	ECPUS	Ha et al. (2008)	20	Unreported		Control difficulty; Persistent need for connection; Specific communication patterns	0.87	N/A	595 Korean high school students (15.9 ± 0.8)	Excessive use	Internet addiction
Mobile Phone Addiction Index	MPAI	Leung (2008)	17	Likert scale	1–5	Inability to control craving; Feeling anxious and lost; Withdrawal/escape; Productivity loss	0.86	N/A	402 Chinese teenagers (16.9 ± unreported)	Addiction	DSM-IV criteria for pathological gambling; Internet addiction; Existing measurement scales of problematic phone use
Questionnaire of Experiences Related to the Cell (Cuestionario de Experiencias Relacionadas con el Movil)	CERM	Beranuy et al. (2009)	10	Likert scale	1–4	Conflicts (related to mobile phone abuse); Problems (due to communicative/emotional use)	0.81	N/A	1879 secondary and undergraduate students (15.5 ± 2.4)	Addiction	DSM-IV criteria for substance abuse and pathological gambling
Cell-Phone Addiction Scale for Korean Adolescents	CPAS	Koo (2009)	20	Likert scale		Withdrawal/tolerance; Life dysfunction; Compulsion/persistence	0.92	N/A	577 Korean adolescents (Unreported)	Addiction	Unreported
Cell-Phone Addiction Assessment Questionnaire	KBUTK	Pawłowska and Potembska (2009)	33	Likert scale	1–5	Salience; Tolerance; Withdrawal; Relapse	0.91	N/A	Adolescent and undergraduate students (Unknown)	Addiction	DSM criteria for pathological gambling
Problem Cellular Phone Use Questionnaire	PCPU-Q	Yen et al. (2009)	12	Dichotomous items	Yes/No	Tolerance; Withdrawal; Negative life consequences; Lack of control	0.85	0.41 - 0.78	10,191 adolescents in Southern Taiwan (14.6 ± 1.8)	Problematic use	DSM-IV-TR criteria for substance use dependence
Questionnaire to Detect New Addictions (Cuestionario de Deteccion de Nuevas Adicciones)	DENA	Labrador Encinas and Villadangos González (2010)	12	Likert scale	0–3	Internet; Video games; Cybercenters; Mobile phone; TV	N/A	N/A	1710 adolescents in Madrid (14.0 ± 1.4)	Addiction	DSM-IV-TR criteria for Substance Abuse disorders
Mobile Phone Involvement Questionnaire	MPIQ	Walsh et al. (2010)	8	Likert scale	1–7	Salience (cognitive/behavioral); Conflict (interpersonal/activities); Relief/euphoria; Loss of control/tolerance; Withdrawal; Relapse and reinstatement	N/A	N/A	946 Australian teenagers and young adults (18.3 ± 2.6)	Involvement	Components model of addiction (Griffiths, 2005)
Mobile Addiction Test	MAT	Martinotti et al. (2011)	10	Likert scale	1–3	Unreported	N/A	N/A	2794 Italian high school students (Unreported)	Addiction	Gambling addiction; Compulsive buying; Internet addiction; Work addiction; Exercise addiction
Smartphone Addiction Proneness Scale	SAPS	Kim et al. (2014)	15	Likert scale	1–4	Disturbance of adaptive functions; Virtual life orientation; Withdrawal; Tolerance	0.81	N/A	795 South Korean adolescents (Unreported)	Adolescent addiction risk	Internet addiction; Mental health problems
Test of Mobile Dependence	TMD	Chóliz (2012)	22	Likert scale	0–4	Abstinence; Lack Control/Problems; Tolerance/Interference	0.94	N/A	2,486 Spanish adolescents (Unreported)	Dependence	DSM-IV-TR definition of the concept of dependence
Text Messaging Gratification Scale	TMG	Grellhesl and Punyanunt-Carter (2012)	47	Likert scale	1–7	Immediate access and mobility; Relaxation/Escape; Entertainment; Information seeking/Coordination; Socialization and affection; Status	0.86	N/A	513 undergraduate students (Unreported)	Gratification with SMS	Uses and Gratification (U and G) Theory (Ruggiero, 2000); Individual needs (Flanagin, 2005)
Problematic Mobile Phone Use Scale	PMPUS	Güzeller and Coñguner (2012)	18	Likert scale	1–5	Interference with negative effect; Compulsion/persistence; Withdrawal/tolerance	0.76–0.83	N/A	950 Turkish high school students (16.1 ± 0.9)	Problematic use	Existing measurement scales for problematic phone use
Mobile Phone Addiction Scale	MPAS	Hong et al. (2012)	11	Likert scale	1–6	Time Management and its Problems; Academic Problems in School and its Influence; Reality Substitute	0.86	N/A	269 Taiwanese female undergraduate students (Unreported)	Addiction	Internet addiction
Smartphone Addiction Inventory	SAI	Kang and Park (2012)	23	Likert scale	1–5	Preoccupation; Daily-life disturbance; Withdrawal; Overuse; Cyber-oriented relationships	0.86	N/A	201 Korean university students (Unknown)	Addiction	Unreported
Mobile Phone Addiction Tendency Scale	MPATS	Xiong et al. (2012)	16	Likert scale	1–5	Withdrawal symptoms; Salience; Social Comfort; Mood changes	0.83	0.91	641 undergraduate students (Unknown)	Addiction	Internet addiction
Smartphone Addiction Scale - Short Version	SAS-SV	Kwon et al. (2013a)	10	Likert scale	1–6	Daily-life disturbance; Positive anticipation; Withdrawal; Cyberspace-oriented relationship; Overuse; Tolerance	0.91	N/A	540 Korean adolescents (14.5 ± 0.5)	Addiction	Existing measurement scales for problematic phone use; Internet addiction
Smartphone Addiction Scale	SAS	Kwon et al. (2013b)	33	Likert scale	1–6	Daily-life disturbance; Positive anticipation; Withdrawal; Cyberspace-oriented relationship; Overuse; Tolerance	0.97	N/A	197 Korean adults (26.1 ± 6.0)	Addiction	Internet addiction; DSM-IV criteria for substance dependence and abuse diagnosis
Problematic Use of Mobile Phones Scale	PUMP	Merlo et al. (2013)	20	Likert scale	1–5	Tolerance; Withdrawal; Longer time than intended; Great deal of time spent using; Craving; Activities given up/reduced; Use despite physical/psychological problems; Failure to fulfill role obligations; Use in hazardous situations; Use despite social/interpersonal problems	0.94	N/A	244 United States adults (29.8 ± 14.1)	Problematic use	DSM-IV criteria for substance abuse; Internet addiction
Self-Rating Questionnaire for Adolescent Problematic Mobile Phone Use	SQAPMPU	Tao et al. (2013)	13	Likert scale	1–5	Withdrawal symptoms; Craving; Physical and mental health status	0.87	N/A	2376 Chinese undergraduate students (Unreported)	Problematic use	Existing measurement scales for problematic phone use
Smartphone Addiction Questionnaire	SPAQ	Al-Barashdi et al. (2014)	39	Unreported		Disregard of harmful consequences; Preoccupation; Inability to control craving; Productivity loss; Feeling anxious and lost	0.76	0.66	140 Sultan Qaboos University undergraduate students (Unreported)	Addiction	Existing smartphone addiction; Casey’s (2012) five-factor smartphone addiction profile
Mobile Phone Use Questionnaire	MP-UQ	King et al. (2014)	29	Dichotomous items	Yes/No	Unreported	N/A	N/A	50 patients with panic disorder; 70 control volunteers (43 ± unreported) (35 ± unreported)	Nomophobia	Unreported
Smartphone Addiction Inventory	SPAI	Lin et al. (2014)	26	Likert scale	1–4	Compulsive behavior; Functional impairment; Withdrawal; Tolerance	0.94	0.74 - 0.91	283 Engineering students from Northern Taiwan (22.9 ± 2.0)	Addiction	Internet addiction
Manolis/Roberts Cell-Phone Addiction Scale	MRCPAS	Roberts et al. (2014)	4	Likert scale	1–7	Withdrawal; More time than expected; Tolerance	0.87	N/A	188 Texas undergraduate students (21 ± unreported)	Addiction	Existing measurement scales for problematic phone use
Mobile Internet Usage Index	MIUI	Shin (2014)	19	Dichotomous items	Yes/No	Excessive use; Neglect of work and social life; Lack of self-control; Use of mobile internet for other reasons than calling	N/A	N/A	Unreported (Unreported)	Dependence	Internet addiction; Existing smartphone addiction measurement scales
Adapted Cell Phone Addiction Test	ACPAT	Smetaniuk (2014)	20	Likert scale	1–5	Preoccupation (salience); Excessive use; Neglecting Work/Social Life; Anticipation; Lack of control	0.93–0.96	N/A	301 United States college students; 362 United States working adults (21 ± unreported) (32 ± unreported)	Addiction	Internet addiction
Adapted Mobile Phone Use Habits	AMPUH	Smetaniuk (2014)	10	Dichotomous items	Yes/No	Salience; Mood modification; Relapse; Withdrawal; Escapism/Dysphoric relief; Tolerance; Cognitive Distortion; Resort to antisocial behavior; Conflict/Loss; Desperation	0.75	N/A	301 United States college students; 362 United States working adults (21 ± unreported) (32 ± unreported)	Symptoms relative to addictive behavior	DSM-IV criteria for pathological gambling
Smartphone Addiction Scale for College Students	SAS-C	Su et al. (2014)	22	Likert scale	1–5	Withdrawal behavior; Salience behavior; Social comfort; Negative effects; Use of application; Renewal of application	0.44–0.88	0.93	243 Chinese college students (unreported)	Addiction	Unreported
Unnamed Nursing Smartphone Addiction Scale		Cho and Lee (2015)	18	Likert scale	1–5	Withdrawal; Tolerance; Interference with daily routines; Positive expectations	0.9	N/A	428 nursing clinical practicum students (Unknown)	Addiction	Internet addiction
Mobile Phone Interference in Life	MPIL	David et al. (2015)	4	Likert scale	1–5	Longer time than intended; Life dysfunction; Loss of control; Loss of productivity	0.81	N/A	992 undergraduate students (19.7 ± 1.9)	Life interference	Unreported
Mobile Phone Problem Use Scale - Short Version	MPPUS-10	Foerster et al. (2015)	10	Likert scale	1–10	Tolerance; Escape from other problems; Withdrawal; Craving; Negative life consequences	0.85	0.40	412 Swiss adolescents (14 ± unreported)	Problematic use	Existing measurement scales for addiction and substance abuse
Phubbing Scale	PS	Karadağ et al. (2015)	10	Likert scale	1–5	Communication disturbance; Phone obsession	0.85–0.87	N/A	401 Turkish university students (21.9 ± unreported)	Phubbing	Focus group interviews
Smartphone Addiction Measurement Instrument	SAMI	Tossell et al. (2015)	15	Likert scale	1–5	Unreported	Unreported	N/A	34 United States undergraduate students (Unreported)	Addiction	Internet addiction; Existing measurement scales for problematic phone use
Problematic Smartphone Use Scale - Revised	PSUS-R	Valderrama (2014)	19	Likert scale	1–6	Salience; Conflict; Tolerance; Withdrawal; Relapse	0.94	N/A	182 United States adults (Unreported)	Problematic use	Components model of addiction (Griffiths, 2005)
Nomophobia Questionnaire	NMP-Q	Yildirim and Correia (2015)	20	Likert scale	1–7	Not being able to communicate; Losing connectedness; Unable to access information; Giving up convenience	0.95	N/A	301 United States college students (20 ± unreported)	Nomophobia	Existing measurement scale for problematic phone use
Untitled Smartphone Addiction Scale		Aljomaa et al. (2016)	80	Likert scale	1–5	Overuse of smartphones; Technological dimensions; Psychological-social dimension; Preoccupation with smartphones; Health dimensions	0.97	0.89–0.92	416 Saudi Arabian university students (Unreported)	Addiction	DSM-IV definition of addiction; Existing measurement scales for problematic phone use
Test of Mobile Dependence - Brief	TMDbrief	Chóliz et al. (2016)	12	Likert scale	0–4	Abstinence; Abuse/interference with activities; Tolerance; Lack of control	0.88	N/A	2028 young adults from Southern and Northwest Europe, South America, India, Pakistan and Mesoamerica (Unreported)	Dependence	Existing measurement scale for problematic phone use
Brief Smartphone Addiction Scale	BSAS	Csibi et al. (2016)	6	Likert scale	1–6	Salience; Mood modification; Tolerance; Withdrawal; Conflict; Relapse	0.82	N/A	441 Hungarian adolescents (13.4 ± 2.2)	Addiction	Existing measurement scale for problematic phone use; Components model of addiction (Griffiths, 2005)
Mobile Addiction Scale	MAS	Fidan (2016)	21	Unreported		Salience; Tolerance; Withdrawal; Relapse; Conflict	0.91	N/A	284 participants from Turkey (Unreported)	Addiction	Components model of addiction (Griffiths, 2005); Mobile Internet tendencies
Mobile Attachment Scale	MAS	Konok et al. (2016)	10	Likert scale	1–5	Phone proximity seeking; Need for contact; Preference for mobile communication	0.77	N/A	142 Hungarian young adults (Unreported)	Attachment-like features of usage	Adult Attachment Scale (AAS; Collins and Read, 1990)
Problematic Mobile Phone Use Scale	PMPUS	Pamuk and Atli (2016)	26	Likert scale	1–5	Deprivation; Adverse outcomes; Control problem; Interaction avoidance	0.92 (EFA); 0.93 (CFA)	0.85	725 college students in Turkey (20.7 ± 0.1)	Problematic use	DSM-5 criteria for SUD and IGD; Existing measurement scale for problematic phone use
Partner Phubbing (Pphubbing) Scale	PPS	Roberts and David (2016)	9	Likert scale	1–5	Unreported	0.92	N/A	308 United States adults (unreported)	Partner phubbing	Personal involvement measure; Relationship satisfaction
Estonian Smartphone Addiction Proneness Scale	E-SAPS 18	Rozgonjuk et al. (2016)	18	Likert scale	1–6	Daily-life disturbance; Cyberspace-oriented relationships; Positive anticipation; Withdrawal and Overuse; Importance; Physical symptoms	0.87	N/A	767 Estonian adults (26.1 ± 6.7)	Addiction proneness	Internet addiction; Existing smartphone addiction measurement scales
Young Adult Attachment to Phone Scale	YAPS	Trub and Barbot (2016)	20	Likert scale	1–7	Refuge (safe with the phone/uncomfortable upon separation); Burden (relief upon separation)	0.94	N/A	955 United States young adults (23.6 ± 2.9)	Attachment	Existing measurement scale for problematic phone use; Attachment anxiety/avoidance
Selfitis Behavior Scale	SBS	Balakrishnan and Griffiths (2017)	20	Likert scale	1–5	Environmental enhancement; Social competition; Attention seeking; Mood modification; Self-confidence; Subjective conformity	0.876	N/A	400 Indian university students (20.9 ± 4.3)	Problematic-self-taking behavior	Focus group interview statements concerning selfitis motivations
Smartphone Application-Based Addiction Scale	SABAS	Csibi et al. (2017)	6	Likert scale	1–6	Tolerance; Withdrawal; Salience; Conflict; Loss of control; Mood modification	0.81	N/A	240 English-speaking volunteers (25.4 ± unreported)	Addiction	Sensation seeking and deprivation sensation; Nomophobia; Existing measurement scales for problematic phone use
Mobile Phone Addiction Craving Scale	MPACS	De-Sola et al. (2017a)	8	Likert scale	1–10	Urgency to use mobile phone; Anxiety due to unavailability	0.92	N/A	1126 Spanish adult mobile phone users (32.8 ± 11.7)	Craving	Existing measurement scales for problematic phone use; State anxiety and impulsivity
Adolescent Preoccupation with Screens Scale	APSS	Hunter et al. (2017)	21	Likert scale	1–6	Mood management; Behavioral preoccupation	0.87–0.91	N/A	1967 Australian adolescents (unreported)	Preoccupation	Existing measurement scales for problematic technology use
Problematic Smartphone Use Scale	PSUS-R	Hussain et al. (2017)	9	Likert scale	1–5	Preoccupation; Withdrawal; Tolerance; Lack of control; Loss of interest in other activities; Overuse despite problems; Deception; Escape/Relieve mood; Social dysfunction	0.86	N/A	640 adult smartphone users (24.9 ± 8.5)	Problematic use	DSM-5 diagnostic criteria for IGD
Smartphone Overuse Screening Questionnaire	SOS-Q	Lee et al. (2017)	28	Likert scale	1–4	Preoccupation; Loss of control; Craving; Insight; Overuse; Neglect of other areas	0.95	0.70	158 subjects from community centers for Internet addiction (22.1 ± 7.6)	Overuse	Existing measurement scale for problematic phone use; Internet addiction
Smartphone Addiction Inventory - Short Form	SPAI-SF	Lin et al. (2017)	10	Likert scale	1–4	Compulsive behavior; functional impairment; Withdrawal; Tolerance	0.84	N/A	268 Engineering students from Northern Taiwan (20.9 ± 1.6)	Addiction	Existing measurement scales for problematic phone use; Proposed diagnostic criteria for smartphone addiction
Mobile Phone Addiction Scale	MPAS	Basu et al. (2018)	20	Likert scale	1–6	Intense desire; Impaired control; Withdrawal; Tolerance; Decreased interest in alternate pleasures; Harmful use	0.90	N/A	388 Indian medical students (20.5 ± 1.8)	Addiction	Existing measurement scales for problematic use
Smartphone Overuse Classification Scale	SOCS	Ding et al. (2018)	24	Likert scale	1–5	Social network app overuse (S-scale); Recreational app overuse (R-scale); Information overload (I-scale)	0.85	0.77–0.88	849 Shanghai university students (Unreported)	Overuse	Internet addiction; Symptoms of psychological dependency
Smartphone Withdrawal Scale	SWS	Eide et al. (2018)	15	Likert scale	1–5	Depression-anxiety; Craving; Irritability-impatience; Difficulty concentration	0.88–0.92	N/A	127 European adults (25.0 ± 4.5)	Withdrawal	Cigarette Withdrawal Scale (CWS; Etter, 2005)
Problematic Mobile Phone Use Questionnaire - Revised	PMPUQ-R	Kuss et al. (2018)	17	Likert scale	1–4	Dependence; Prohibited use; Dangerous use	0.86	N/A	512 United Kingdom young adult smartphone users (25.5 ± unreported)	Problematic use	Existing measurement scales for problematic phone use; Psychopathology (depression, anxiety, stress, ADHD)
Problematic Mobile Phone Use Questionnaire – Short Version	PMPUQ-SV	Lopez-Fernandez et al. (2018)	15	Likert scale	1–4	Dependence; Prohibited use; Dependence	0.69–0.88	N/A	3038 adults from 14 different countries (26.5 ± 9.4)	Problematic use	Existing measurement scales for problematic use
Questionnaire to Assess Nomophobia	QANIP	Olivencia-Carrion et al. (2018a)	11	Unreported		Mobile Phone Abuse; Loss of Control; Negative Consequences; Sleep Interference	0.80	N/A	968 Spanish adults (23.2 ± 7.2)	Nomophobia	Unreported
Cuestionario de Abuso del Telefono Movil	ATeMo	Olivencia-Carrion et al. (2018b)	25	Likert scale	0–4	Craving; Loss of Control; Negative Life Consequences; Withdrawal Syndrome	0.91	N/A	856 Spanish university students (21.1 ± 3.1)	Abuse	Gambling disorder; Substance abuse disorders; Existing measurement scales for problematic phone and Internet use
MULTICAGE-TIC		Pedrero-Pérez et al. (2018)	20	Dichotomous	Yes/No	Problematic use of: Internet, video games, mobile phones, instant messaging, social networks	0.72–0.93	N/A	1276 Spanish-speaking adults (unreported)	Problematic use	MULTICAGE CAD-4 screener for compulsive behaviors (Pedrero-Pérez et al., 2007)
Problematic Media Use Measure	PMUM	Domoff et al. (2019)	27	Likert scale	1–5	Unsuccessful control; Loss of interest; Preoccupation; Psychosocial consequences; Serious problems due to use; Withdrawal; Tolerance; Deception; Escape/Relieve mood	0.97	N/A	291 mothers of children aged 4–11 (Unreported)	Parent-report of adolescent problematic media use	DSM-5 criteria for IGD
Problematic Media Use Measure - Short Form	PMUM-SF	Domoff et al. (2019)	9	Likert scale	1–5	Unsuccessful control; Loss of interest; Preoccupation; Psychosocial consequences; Serious problems due to use; Withdrawal; Tolerance; Deception; Escape/Relieve mood	0.93	N/A	632 mothers of children aged 4–11 (40.4 ± 10.0)	Parent-report of adolescent problematic media use	DSM-5 criteria for IGD
Parental Smartphone Use Management Scale	PSUMS	Hsieh et al. (2019)	17	Likert scale	0–6	Reactive management; Proactive management; Monitoring	0.93–0.95	N/A	237 parents of adolescents with ADHD (Parents: 43.5 ± 5.9) (Adolescents: 13.7 ± 1.8)	Parent’s self-efficacy	Existing measurement scale for problematic use
Smartphone Impact Scale	SIS	Pancani et al. (2019)	26	Likert scale	1–5	Loss of control; Nomophobia; Smartphone-mediated communication; Emotion regulation; Support to romantic relationships; Task support; Awareness of negative impact	0.74–0.91 (ω)	N/A	601 Italian adults (29.1 ± 9.3)	Impacts of use	Existing measurement scale for problematic use

**TABLE 2:** Smartphone use frequency scales.

Title	Abbrev.	Author(s)	Items	Item format	Item scale	Content domains	Internal consistency (Cronbach’s α)	Temporal stability (Test-Retest)	Sample/Norms (Age: *M*, *SD*)	Purported construct	Criterion-Related validity
Media and Technology Usage and Attitudes Scale	MTUAS	Rosen et al. (2013)	60	Likert scale	1–5	Smartphone usage; Social media usage; Internet searching; E-mailing; Media sharing; Text messaging; Video gaming; Online friendships; Facebook friendships; Phone calling; Watching TV; Positive/Negative attitudes; Tech anxiety/dependence; Attitudes toward task-switching	0.61–0.97	N/A	942 United States adults (30.0 ± 12.5)	Involvement	Internet addiction; Technology-related anxiety; Daily media usage hours
Smartphone Use Frequency	SUF	Elhai et al. (2016)	11	Likert scale	1–6	Calling; Messaging; Email; Social networking; Internet; Gaming; Music/podcast; Taking pictures/videos; Watching videos; Reading; Navigation	0.86	N/A	308 North American adults (33.2 ± 10.2)	Usage	Unreported
Mobile Usage Scale	MUS	Konok et al. (2016)	6	Likert scale	1–5	Smart mobile phone use; Traditional mobile phone use	0.71	N/A	142 Hungarian young adults (Unreported)	Mobile usage types	Mobile phone use

**TABLE 3:** Smartphone use motivations and attitudes scales.

Title	Abbrev.	Author(s)	Items	Item format	Item scale	Content domains	Internal consistency (Cronbach’s α)	Temporal stability (Test-Retest)	Sample/Norms (Age: *M*, *SD*)	Purported construct	Criterion-Related validity
Attitudes Toward Cell Phones Questionnaire	ATCPQ	Aoki and Downes (2003)	40	Likert scale	1–7	Necessity in Modern Times; Cost Efficiency; Safety/Security; Dependency; Negatives; Functionality	0.81	N/A	137 undergraduate students (Unreported)	Attitudes toward phones	Unreported
Mobile Phone Usage Scale	MPUS	Hooper and Zhou (2007)	30	Likert scale	1–5	Behaviors: Habitual; Addictive; Mandatory; Voluntary; Dependent; Compulsive	0.53–0.88	N/A	184 undergraduate students (Unreported)	Motivations of usage	Existing measurement scale for problematic phone use
Media and Technology Usage and Attitudes Scale	MTUAS	Rosen et al. (2013)	60	Likert scale	1–5	Smartphone usage; Social media usage; Internet searching; E-mailing; Media sharing; Text messaging; Video gaming; Online friendships; Facebook friendships; Phone calling; Watching TV; Positive/Negative attitudes; Tech anxiety/dependence; Attitudes toward task-switching	0.61–0.97	N/A	942 United States adults (30.0 ± 12.5)	Involvement	Internet addiction; Technology-related anxiety; Daily media usage hours
Gravitating Toward Mobile Phone Scale	GoToMP	Olufadi (2015)	38	Likert scale	1–4	Boredom; Social connection; Class-related use; Emergency; Addiction; Perceived behavioral control	0.94	N/A	432 Nigerian undergraduate students (21.1 ± 2.1)	Urge to use during lectures	Theory of Consumption Values (TCV; Sheth et al., 1991)
Process vs Social Smartphone Usage Scale	PSSU	van Deursen et al. (2015)	12	Likert scale	1–5	Process usage motivations; Social usage motivations	0.89	N/A	386 Dutch adolescents and adults (35.2 ± 14.7)	Motivations	Perceived gratification items (Chua et al., 2012); Uses and Gratification (U and G) Theory (Ruggiero, 2000)
Mobile Phone Affinity Scale	MPAS	Bock et al. (2016)	24	Likert scale	1–5	Connectedness; Productivity; Empowerment; Anxious attachment; Addiction; Continuous Use	0.83	N/A	1058 North American adults (32.5 ± 10.3)	Affinity	Anxiety and impulsiveness; Psychological resilience

### Description of Measurement Scales

Following the development of the first mobile phone use measurement scales (Toda et al., 2004), mobile phone ownership began decreasing as smartphones became increasingly more popular. However, during this transition from mobile phone use to the “smartphone era,” the terms “mobile phone” and “smartphone” were used interchangeably across studies and, often, within studies. Because smartphones have significantly more components and capabilities than mobile phones and the problematic use of the two different forms of technology should be viewed differently, comparisons of published scales should be made in light of the distinctive differences between the two types of technology. The scales are arranged based upon the date that they were published within each of the three tables starting with the first developed scale in 2004 to the most recently published scales in 2019. Thus, it is likely that the more recently developed scales involved specific analyses of smartphone use and behavior as opposed to that of more contemporary mobile phone devices.

Scales included in Table 1 are those that were specifically developed and validated to identify problematic smartphone or mobile phone use or to diagnose individuals with smartphone addiction, overuse, dependency, attachment, etc. [e.g., Smartphone Addiction Scale (SAS; Kwon et al., 2013b); Smartphone Addiction Inventory (SPAI; Lin et al., 2014)]. Although the construct being claimed to be measured by each of these individual scales may differ, many of them are similar in their theoretical foundations and even item content. For example, while Kwon et al. (2013b) utilized DSM-IV criteria for substance dependence to develop the item content for the SAS with the goal of assessing “addiction,” Merlo et al. (2013) utilized the same criteria to develop the Problematic Use of Mobile Phones (PUMP) scale. In Table 1, validated shortened versions of originally validated scales were included in the review if identified in the literature review. Table 1 includes the majority of the scales identified in the review (70 of the 78).

Table 2, on the other hand, contains three scales. This table includes scales assessing smartphone use frequency as opposed to general problematic smartphone use behavior. It is important to note the differences between these two constructs. As described earlier, smartphone use frequency can be very heterogeneous due to differing motivations and purposes for use (Elhai et al., 2018). Higher frequency of smartphone use may not indicate the presence of problematic smartphone use if, for example, associated life dysfunction is not identified (Billieux, 2012). Scales included in this table include: the Media and Technology Usage and Attitudes Scale (MTUAS; Rosen et al., 2013) which assesses both use of technological devices – including smartphones – and attitudes surrounding technology use (see Table 3); the Smartphone Use Frequency (SUF; Elhai et al., 2016) assessing use of smartphone devices in areas such as calling, messaging, emailing, etc.; and the Mobile Usage Scale (MUS; Konok et al., 2016) examining differences in use of smartphones and traditional mobile phones. These scales were included in the review so as to provide researchers with options for examining problematic smartphone use and/or smartphone use frequency.

Finally, additional scales assessing motivations for as well as attitudes surrounding use of smartphone devices are included in Table 3. For example, the MTUAS (Rosen et al., 2013) is included in both Tables 2, 3 because, although it assesses frequency of smartphone and technology usage, it also examines attitudes associated with this usage. Additionally, the Process vs Social Smartphone Usage Scale (PSSU; van Deursen et al., 2015) was included in Table 3 due to its examination of motivations for use of smartphone devices. Finally, a third example of a scale included in Table 3 is the Mobile Phone Affinity Scale (MPAS; Bock et al., 2016) which evaluates motivations of smartphone use, including connectedness, productivity, empowerment, etc. It was important to include these six scales in Table 3 because their inclusion further exemplifies the robust nature of the research and development of measurement scales focusing on smartphone use. The inclusion of each of these three domains makes this review a useful tool for researchers studying smartphone use behavior – problematic or otherwise – as well as associated benefits and dysfunction.

### Psychometric Characteristics

Elements of criterion-related validity, content domains, internal consistency, temporal stability, and purported construct were listed or briefly described for each of the scales in Tables 1–3. Because of this, these tables can be used to compare the individual scales. Additionally, in the following analysis, the psychometric properties and conceptual foundations of the scales included in this review will be further dissected. This analysis will help researchers and practitioners alike to consider the psychometric properties and theoretical foundations of potential assessment tools before deciding which scale should be utilized in their research or practice.

The term “addiction” was used frequently when naming many of the problematic smartphone use scales. This is due to the choice of framework and criterion-related validity used when developing and validating the scales. Many scale developers used either the DSM-IV or DSM-5 criteria for substance use to examine criterion-related validity during development. Others chose to use Griffiths’ (2005) components descriptive model of addiction, which includes the following core components: salience, mood modification, tolerance, withdrawal, conflict and relapse. Similarly, Internet addiction was frequently used to establish criterion-related validity. Before the release of the first smartphone, problematic Internet use was being observed, identified, and subsequently labeled as Internet addiction. Addiction scales were quickly developed to assist identifying this behavior such as Young’s Internet Addiction Test (IAT or Y-Scale; Young, 1998). Once smartphones were developed and made available to the public, problematic smartphone use similarly became a concern. Many researchers utilized various Internet addiction scales to validate their scales (e.g., SMS-PUDQ; ECPUS; MPAI; SAPS).

One of the final ways that scale developers established criterion-related validity was by utilizing quantified smartphone use as a criterion to determine whether the scales could be used to identify smartphone addiction. However, most of these scale development processes involved self-reported and self-estimated smartphone use. Because they were unable to utilize concrete and exact documentation of participants’ smartphone use time, they relied upon estimation which can be unreliable (Andrews et al., 2015) and, therefore, should not be considered to be a practical or accurate means of validating a scale.

Even if accurate data were being obtained from participants concerning time spent using their phones, there is no established cut-off point that can be used to validate accuracy of a scale in indicating dependency, problematic use or addiction based upon extensiveness of use alone since it has not been determined at what point phone use becomes problematic. It is likely that a cut-off point for quantitative smartphone use may not be feasible. Elhai et al. (2018) explains that smartphone use frequency can be very heterogeneous due to differing motivations and purposes for use. They describe how a high frequency of smartphone use can be functional for some (e.g., productive smartphone use for purposes of work or school) and dysfunctional for others (e.g., excessive gaming and social media use).

Additionally, a significant number of scales described in Tables 1–3 relied upon existing measurement scales for problematic phone use in order to establish concurrent validity for the scale they were developing. This is due to the recognized issue of currently not having a gold standard for criterion-related validity for problematic phone use or addiction. However, this is concerning considering the existing assessments used to validate the new scale likely also used problematic criteria to establish criterion-related validity. For example, when developing the Smartphone Impact Scale (SIS), Pancani et al. (2019) included the widely used Smartphone Addiction Scale (SAS; Kwon et al., 2013b) in their study to validate the SIS with an Italian adult sample. This could be problematic for two reasons. Firstly, to our knowledge, the SAS has yet to be validated for use with Italian adults as it was developed using a population of Korean adults. Secondly, to our knowledge, the temporal stability of the SAS has yet to be investigated.

Selection of content domains by the researchers in their validation studies stemmed from their conceptual foundation for the scale’s development and their criterion-related validity. For example, regarding the scales in Table 1, DSM-IV pathological gambling criteria or DSM-5 gambling disorder criteria were used to establish criterion-related validity for seven of the scales (COS, MPAI, CERM, KBUTK, MAT, AMPUH, and ATeMo). Therefore, these scales’ content domains were shown to reflect the diagnostic criteria associated with problematic gambling disorder. The DSM-5 indicates that, to be diagnosed with gambling disorder, an individual must exhibit four or more of the following symptoms: tolerance; withdrawal; lack of control; preoccupation; escape from problems; “chasing” losses; deception; and associated life dysfunction in areas such as relationships, job, education, or finances (American Psychiatric Association, 2013). Excluding “chasing” losses, these factors were shown to be consistently reflected across those seven scales in terms of their established content domains. DSM-IV, DSM-IV-TR or DSM-5 criteria for SUDs were frequently used to validate these problematic smartphone use scales, and their diagnostic criteria were similarly, reflected in the content domains established in the validation studies (e.g., CERM, PCPU-Q, and SAS).

Internal consistency is the degree of interrelatedness among scale items (Mokkink et al., 2013). This measure of reliability was reported for most of the scales in the form of a Cronbach’s α value. However, despite its importance in scale development, an internal consistency value was not reported for seven of the scales in their validation studies (STDS, DENA, MPIQ, MAT, MP-UQ, MIUI, and SAMI). The Cronbach’s α values that were reported ranged from 0.53 (MPUS) to 0.97 (PMUM, MTUAS, and SAS). Although there is inconsistency in the field regarding at what point Cronbach’s α values should be considered to be adequate or acceptable, acceptable values of alpha have been reported to range from 0.70 to 0.95 (Nunnally and Bernstein, 1994; Bland and Altman, 1997; DeVellis, 2016). Using the lowest value reported as being acceptable or adequate as a cutoff, four of the scales identified in this review (PMPUQ, IMAT, MPUS, and MTUAS) would not meet that standard.

Although internal consistency is important in scale development, most of the scale developers failed to account for temporal stability in guaranteeing reliability. Upon analyzing the psychometric properties of the various scales, it was discovered that only in the scale development of ten scales were test-retest reliability coefficients provided to indicate that the scales have temporal stability. This is a cause for concern because even some of the most frequently used scales have failed to ensure temporal stability in their development (e.g., SAS, NMP-Q, and SABAS).

## Discussion

This review is the first to identify and report the method of development for all problematic smartphone use scales as well as those developed to assess smartphone use frequency, motivations, and attitudes. After conducting a systematic search and identifying all relevant measures, we analyzed the psychometric properties and criterion-related validity of each scale. However, despite identifying 78 validated scales, we were not able to fully determine the most efficient scales for measuring problematic phone usage due to several issues: (1) most of the scales established criterion validity using DSM-IV or DSM-5 criteria for gambling disorder or substance-use disorders, even though there is still considerable controversy over whether problematic smartphone use should be considered an “addiction”; (2) test-retest reliability coefficients were not reported in the development articles for 68 of the 78 scales, and both internal consistency *and* test-retest reliability were not available for seven of the scales, which causes concern for future analyses that attempt to identify the most efficient scale(s); (3) the gold-standard criteria and cut-off scores for problematic smartphone use has yet to be established; in other words, these scales cannot accurately be compared and contrasted since there is no validated, gold-standard criteria to which they can strive to incorporate. Therefore, we will primarily discuss practical ideas and recommendations for future research.

### Scale Content

The addition of gambling disorder to the substance-related and addictive disorders section of the DSM-5 as a non-substance-related addictive disorder has subsequently opened the door for other behaviors to be researched, evaluated, and identified through developed and validated scales. Another example of this would be the behavioral condition known as internet gaming disorder (IGD). While this area of research warrants further study according to the DSM-5 (American Psychiatric Association, 2013), the proposed criteria for IGD as a behavioral addiction involving the problematic use of video game technology closely resembles the criteria for SUD and are very similar to how researchers are conceptualizing problematic smartphone use (Lin et al., 2016). Further, based on the development methods of the majority of the reviewed scales, many researchers feel as though it could be time to start assessing smartphone use with an addictive framework in mind, arguably with the exception of “tolerance” symptoms (Lin et al., 2016, 2017). This may in part be due to a belief that problematic smartphone use, as well as potentially other problematic behaviors, should be similarly characterized and defined as diagnosable behavioral or non-substance-related addictions. The majority of reviewed scales reflect this viewpoint. The content domain of most scales (see Table 1) are related to dependence-related concepts including craving, tolerance, withdrawal, excessive time spent using, and negative life consequences.

Other scales have moved away from this content domain in their development and have attempted to measure more specific and different aspects of problematic smartphone use. For example, The Mobile Phone Involvement Questionnaire (Walsh et al., 2010) and the Media and Technology Usage and Attitudes Scale (Rosen et al., 2013) examine smartphone use involvement through items assessing euphoria, salience, and overall usage. This perhaps reflects the rationale that smartphones may be especially cognitively and behaviorally salient to some, resulting in more usage, but without this usage necessarily being pathological, uncontrollable, or addictive in nature. Such scales perhaps measure the construct of “liking,” or the pleasurable impact of habitual smartphone use, compared to other scales measuring the construct of “wanting,” or the compulsive motivation to engage in smartphone use resulting in negative life consequences. This reflects an important distinction considering the behavioral addiction framework: more and more in today’s society, smartphones are linked with several forms of reward and social value. It makes sense people would “like” smartphones, feel they are important, and use them many times a day. This does not necessarily reflect addiction to them, despite individual’s tendency to self-report this. Some of the reviewed scales perhaps are better conceptualized as a measuring maladaptive smartphone use, rather than addictive use, as endorsements such behaviors perhaps do not rise to the severity levels of addiction (Panova and Carbonell, 2018).

In a similar vein, some scales appear to measure the degree to which individuals report salient emotional connections to their smartphone. The Young Adult Attachment to Phone Scale (Trub and Barbot, 2016) and the Adolescent Preoccupations with Screens Scale (Hunter et al., 2017) share item content related to feelings of safety with and feelings of anxiety when without one’s phone. Such scales measure attachment styles, in that an individual’s mood state can shift depending on the smartphone device’s proximity. The relative convenience of smartphone functions in daily life can mean that feelings of irritation or concern are likely to present when one does not have immediate access to it. Relatedly, scales like the Mobile Attachment Scale (Konok et al., 2016) and the Mobile Phone Affinity Scale (Bock et al., 2016) have items which measure a preference for mobile communication, resulting in strong preferences for having one’s smartphone device instantly accessible. This emotional attachment resulting in dysphoria can mimic addiction withdrawal symptoms in this way. Problematic smartphone use often co-occurs with depression and anxiety as a means of experiential avoidance (Elhai et al., 2017). But, these scales and criteria may simply be reflecting a strong “liking” for the ease of communication to others via calling/texting, can result in different emotional reactions depending on whether the device is accessible or not. Future research should examine how endorsement of particular problematic smartphone use behaviors perhaps better explained by general psychopathology like depression and anxiety, rather than addiction.

Numerous researchers have published scales purportedly assessing “smartphone addiction” or “phone addiction.” However, some researchers feel as though we do not currently have the necessary evidence supported by research to accurately conceptualize smartphone use as having the capability of developing into an addictive behavior. Griffiths (2013) argues that “we are not yet in a position to confirm the existence of a serious and persistent psychopathological addictive disorder related to mobile phone addiction on the basis of population survey data alone” (p. 77). Perhaps this is the reason that a standard cut-off point to determine when smartphone use becomes problematic has yet to be established. Similarly, the Internet addiction framework was frequently used by the developers of several of the reviewed problematic smartphone use scales to establish criterion-related validity. However, Internet addiction is not currently recognized by the DSM-5 as a non-substance related addictive disorder due to the lack of research indicating similarity in manifestation or dysfunction with addictive disorders recognized by the DSM-5.

Additionally, there is a lack of sufficient research investigating how to effectively characterize problematic smartphone use, and it is currently unclear whether “problematic” ought to be defined by the quantity of use, patterns of use, or by the negative consequences or marked distress as a result of usage. If researchers intend to define problematic smartphone use as an “addiction” similar to a substance-use disorder, all three of those criteria, among others (e.g., “recurrent use in situations in which it is physically hazardous” or “continued use despite having persistent or recurrent social or interpersonal problems caused by or exacerbated by use”), would need to be present in order to diagnose dysfunctional or problematic smartphone use as an addiction (American Psychiatric Association, 2013). This should be reflected in the self-report scales researchers are developing, testing, and validating.

Panova and Carbonell (2018) support Griffiths’ (2013) previously described argument in that they similarly suggest moving away from the addiction framework when considering problematic behaviors such as the problematic use of smartphone or other technological devices. They reference a pattern of weak study designs in the smartphone literature, such as full reliance on correlational studies, or a lack of longitudinal and experimental studies that examine associated cognitive, psychological or behavior dysfunction. They also strongly advocate for the use of terms such as “problematic use” over “addiction” when describing these behaviors. They noted that it is imperative that a research-supported criterion for problematic smartphone use be identified before using officially recognized addictive disorders to establish criterion-related validity.

### Limitations of Reviewed Scales

In addition, there were many fundamental limitations to the development and intended uses of the reviewed scales. For instance, all of the reviewed scales that assessed phone use were self-report, and, therefore, cannot reliably measure actual phone usage. This is a limitation in this particular field of research that needs to be addressed. Further, when developing these new scales, many of the researchers’ hypotheses for creating these scales were that problematic phone use would correlate not with actual use, but, instead, with associated personality traits including self-esteem and impulsivity (e.g., Bianchi and Phillips, 2005; Billieux et al., 2008; Leung, 2008). Future research may aim to develop or modify an existing scale or consider running an experimental study in which they actively measure phone usage among individuals that includes a method to separate “normal” and “problematic” use. Interestingly, global researchers (Monge Roffarello and De Russis, 2019), as well as Google (2019), have recently created smartphone applications (e.g., “Socialize” and “Digital Wellbeing”) that can track phone usage among other features and even provide an intervention for excessive use (e.g., allowing users to set limits for amount of time allotted for specific application usage per week or per day). These applications are excellent examples for researchers to consider using as an alternative to self-report scales in measuring smartphone usage.

In a research setting, these applications would provide investigators with the opportunity to gather objective data on smartphone use from smartphone-using participants following the instruction of having the application downloaded on participants’ phones for a specific period of time. Future research ought to also investigate the effectiveness of these applications as intervention tools for problematic smartphone use. However, to reiterate, due to the heterogeneity of smartphone use frequency (i.e., functional versus dysfunctional) described by Elhai et al. (2018), researchers should recognize that objective smartphone use data collected through use of these applications or other methods is a measure of smartphone use frequency, not necessarily problematic smartphone use. Additionally, necessary steps ought to be taken to safeguard ethical considerations and minimize risks associated with instructing participants to download applications on their smartphone devices with the inherent function of tracking their activity (e.g., protection of privacy).

Secondly, the majority of the development articles for the reviewed scales reported only internal consistency as a means of establishing reliability for their scale. Internal consistency is widely used in scale development, and the coefficient is based off of the interrelatedness of the items within the scale. However, this does not mean that the items as a whole are necessarily related to the intended construct or possess established validity. If we were going to rank scales as the most reliable based solely off of their internal consistency coefficients, the PMUM, MTUAS, and SAS (α = 0.97) would have been at the top of the list. However, researchers such as Thompson (2003) have called the use of internal consistency as the sole measure of reliability “sloppy” and not representative of the quality of the scale. While a higher Cronbach’s alpha may demonstrate the consistency of the items in the measure, the items may not be accurately capturing problematic phone usage. If there had been other reliability and validity statistics offered in the development articles of the reviewed scales, perhaps specific scales could have been recommended with confidence in this review for future use.

Thirdly, there was a large amount of variability in the types of samples studied in these development articles, which makes it difficult to compare the utility of each scale. The following should be interpreted not as limitations, but, instead, as interesting findings about the diverse origin of subjects studied in each scale’s development. For instance, a small percentage (16/84) of the studies were conducted with participants in the United States, with many of the scales having been written in languages besides English. For example, as many as six scales were developed in Chinese (WEUS, PSUMS, MPAI, SAS-C, SQAPMPU, and MPATS), four in Turkish (MAS, PMPUS, PS, and PMPUS), and seven in Korean (ECPUS, CPAS, SAPS, SAI, SAS, SAS-SV, and SOS-Q). Based on research conducted by the Pew Research Center (2018), South Korea has the largest percentage of smartphone owners. Therefore, the large number of scales that have been developed for and within that population is understandable. Yet, there were also several scales that were developed in English-speaking areas outside of the United States, such as in Australia (MMPUS, IMAT, APSS, and MPIQ) and the United Kingdom (PMPUQ-R). All of this information can be viewed in Tables 1–3 and Appendix A.

Lastly, the intended use of the reviewed scales varied depending on the theoretical models or criteria upon which they were based. Most of the scales were intended to measure problematic use, addiction, dependence, and excessive use of mobile phones. For instance, Leung (2008), one of the earliest developers of a mobile phone index used to measure “addiction” symptoms demonstrated by mobile users, based her construction of the MPAI off of the idea that adolescents had started excessively using mobile phones during their leisure time as a way of counteracting boredom due to too much time with not enough to do; further, this type of activity, labeled as “leisure boredom,” had been shown to be associated with deviant activity and negative affect. Interestingly, there was a large percentage (38%) of scales purportedly assessing smartphone or mobile phone “addiction,” which is surprising given the aforementioned literature that has been opposed to labeling problematic smartphone use as an “addiction” (Griffiths, 2013; Panova and Carbonell, 2018). Additionally, while several of these scales were developed with the hopes of being used in the future for clinical purposes (e.g., diagnosis of problematic smartphone use), since there is no mention of problematic smartphone use as a disorder an addiction in the DSM-IV or DSM-5 or ICD-11, it seems as though authors must become content with their scales being confined for research purposes only. This further indicates a need for additional research on the conceptualization and demonstrated severity of problematic smartphone use, and whether it should be given consideration for a place in the next edition of the DSM or ICD. Until then, we are unable to recommend the use of a specific scale or specific scales to assess this behavior due to a lack of sufficient research on the construct.

### Limitations of the Current Study

This review is not without limitations. First, the only databases used in the systematic search were PsycINFO (EBSOhost) and MEDLINE Complete (EBSCOhost). PsycINFO was utilized due to being a specialized database that can provide unique search results specific to topics of psychology; additionally, it has been used in several largely cited systematic reviews (Bramer et al., 2017; Elhai et al., 2017). We also utilized the MEDLINE Complete database rather than the PubMed interface due to the convenience of access through EBSOhost. Secondly, many reliability coefficients were not able to be listed due to many of the articles being published solely in a foreign language and, therefore, we were unable to identify and/or interpret the coefficients; the articles being inaccessible; or simply because the coefficients were not reported in the articles. That last point may also be expressed as a limitation of the scales themselves.

### Future Directions

Future research must be conducted in order to further identify potential cognitive, neurological, physical, behavioral or social dysfunction related to smartphone use. Currently, no causal relationships between smartphone use and dysfunction in these previously listed areas have been established. Until then, conceptualizing smartphone use in such a way as to assert that the behavior can become problematic or clinical in nature should be done with caution. Additionally, a standard cut-off point at which smartphone use becomes dysfunctional ought to be investigated. With more evidence of causal relationships between smartphone use and dysfunction as well as a more formulated and standardized conceptualization of the behavior, researchers will be able to construct more accurate and specific scales for identifying problematic use.

## Conclusion

This review serves as an opportunity to compare and contrast the numerous scales that have been published in the past 13 years and to analyze the psychometric properties of each of the individual scales in order to determine which, if any, of the included scales should be considered to be adequate tools for assessing problematic smartphone use or smartphone addiction. However, it is recommended that further research be conducted to sufficiently conceptualize the behavior and its development, manifestation, and associated dysfunction. In order to best develop tools to assess the behavior, we must first understand smartphone use with an increased focus on contexts, functions, and motivations for use, rather than simply borrowing item criteria from assessment scales of more established substance or behavioral addictions. Currently, there is still much to learn about smartphone use and at what point and for whom the use becomes problematic.

## Author Contributions

BH and SF conceived the study, interpreted the data, and participated in drafting the manuscript. TR and JS participated in drafting the manuscript. All authors read and approved the final manuscript.

## Conflict of Interest

The authors declare that the research was conducted in the absence of any commercial or financial relationships that could be construed as a potential conflict of interest.

## Appendix A

### EBSCOhost Search Strategy

(smartphone[tiab] OR smart phone[tiab] OR cellular phone[tiab] OR cell phone[tiab] OR cell-phone[tiab] OR mobile device[tiab] OR mobile phone[tiab])

AND

(problematic[tiab] OR problem[tiab] OR dependence[tiab] OR dependency[tiab] OR overuse[tiab] OR addiction[tiab] OR nomophobia[tiab] OR attachment[tiab] OR excessive[tiab] OR compulsive[tiab])

AND

(questionnaire[tiab] OR scale[tiab] OR index[tiab] OR test[tiab] OR inventory[tiab] OR index[tiab] OR instrument[tiab] OR assessment[tiab] OR measurement[tiab] OR survey[tiab] OR psychometric^∗^[tiab] OR validation[tiab] OR development[tiab])

AND

(“1990”[PDAT]: “2019”[PDAT]).

## References

[B1] Al-Barashdi H. Bouazza A. Al Zubaidi A. (2014). Psychometric properties of smartphone addiction questionnaire (SPAQ) among sultan qaboos university undergraduate students. *J. Educ. Psychol. Stud.* 8 637–644. 10.12816/0014333

[B2] Aljomaa S. Al Qudah M. Albursan I. Bakhiet S. Abduljabbar A. (2016). Smartphone addiction among university students in the light of some variables. *Comput. Hum. Behav.* 61 155–164. 10.1016/j.chb.2016.03.041

[B3] Alosaimi F. D. Alyahya H. Alshahwan H. Al Mahyijari N. Shaik S. A. (2016). Smartphone addiction among university students in Riyadh, Saudi Arabia. *Saudi Med. J.* 37 675–683. 10.15537/smj.2016.6.14430 27279515 PMC4931650

[B4] American Psychiatric Association (1995). *Diagnostic and Statistical Manual Of Mental Disorders: DSM-IV.* Washington, DC: American Psychiatric Association.

[B5] American Psychiatric Association (2000). *Diagnostic and Statistical Manual Of Mental Disorders: DSM-IV-TR.* Washington, DC: American Psychiatric Association.

[B6] American Psychiatric Association (2013). *Diagnostic and Statistical Manual Of Mental Disorders*, 5th Edn Washington, DC: American Psychiatric Association.

[B7] American Psychiatric Association (2017). *What is Addiction?.* Washington, DC: American Psychiatric Association.

[B8] Andrews S. Ellis D. A. Shaw H. Piwek L. (2015). Beyond self-report: tools to compare estimated and real-world smartphone use. *PLoS One* 10:e0139004. 10.1371/journal.pone.0139004 26509895 PMC4625000

[B9] Aoki K. Downes E. J. (2003). An analysis of young people’s use of and attitudes toward cell phones. *Telemat. Inform.* 20 349–365.

[B10] Apple Inc (2007). *Apple Reinvents the Phone with iPhone.* Available online at: https://www.apple.com/newsroom/2007/01/09Apple-Reinvents-the-Phone-with-iPhone/ (accessed July 26, 2018).

[B11] Balakrishnan J. Griffiths M. D. (2017). An exploratory study of “Selfitis” and the development of the selfitis behavior scale. *Intern. J. Ment. Health Add.* 16 722–736. 10.1007/s11469-017-9844-x 29904329 PMC5986832

[B12] Basu S. Garg S. Singh M. M. Kohli C. (2018). Addiction-like behavior associated with mobile phone usage among medical students in Delhi. *Indian J. Psychol. Med.* 40 446–451. 10.4103/IJPSYM.IJPSYM_59_18 30275620 PMC6149311

[B13] Beranuy M. Chamarro A. Graner C. Carbonell X. (2009). Validación de dos escalas breves para evaluar la adicción a Internet y el abuso de móvil. *Psicothema* 21 480–485. 19622333

[B14] Bianchi A. Phillips J. G. (2005). Psychological predictors of problem mobile phone use. *Cyberpsychol. Behav.* 8 39–51. 10.1089/cpb.2005.8.39 15738692

[B15] Billieux J. (2012). Problematic use of the mobile phone: a literature review and a pathways model. *Current Psychiatry Reviews* 8 299–307. 10.2174/157340012803520522

[B16] Billieux J. Schimmenti A. Khazaal Y. Maurage P. Heeren A. (2015). Are we overpathologizing everyday lift A tenable blueprint for behavioral addiction research. *J. Behav. Add.* 4 119–123. 10.1556/2006.4.2015.009 26014667 PMC4627665

[B17] Billieux J. Van der Linden M. Rochat L. (2008). The role of impulsivity in actual and problematic use of the mobile phone. *Appl. Cogn. Psychol.* 22 1195–1210. 10.1002/acp.1429

[B18] Bland J. Altman D. (1997). Statistics notes: cronbach’s alpha. *BMJ* 314:275.

[B19] Bock B. C. Lantini R. Thind H. Walaska K. Rosen R. K. Fava J. L. (2016). The mobile phone affinity scale: enhancement and refinement. *JMIR Mhealth Uhealth* 4:e134. 10.2196/mhealth.6705 27979792 PMC5200845

[B20] Bramer W. M. Rethlefsen M. L. Kleijnen J. Franco O. H. (2017). Optimal database combinations for literature searches in systematic reviews: a prospective exploratory study. *Syst. Rev.* 6:245. 10.1186/s13643-017-0644-y PMC5718002 29208034

[B21] Casey B. M. (2012). *Linking Psychological Attributes to Smartphone Addiction, Face-to-Face Communication, Present Absence and Social Capital.* Unpublished Master’s thesis. The Chinese University of Hong Kong, Hong Kong, China.

[B22] Chen J. Liang Y. Mai C. Zhong X. Qu C. (2016). General deficit in inhibitory control of excessive smartphone users: evidence from an event-related potential study. *Front. Psychol.* 7:511. 10.3389/fpsyg.2016.00511 27148120 PMC4830824

[B23] Cho S. Lee E. (2015). Development of a brief instrument to measure smartphone addiction among nursing students. *Comput. Inform. Nurs.* 33 216–224. 10.1097/CIN.0000000000000132 25636040

[B24] Chóliz M. (2012). Mobile-phone addiction in adolescence: the test of mobile phone dependence (TMD). *Prog. Health Sci.* 2 33–44.

[B25] Chóliz M. Pinto L. Phansalkar S. S. Corr E. Mujjahid A. Flores C. (2016). Development of a brief multicultural version of the test of mobile phone dependence (TMDbrief) questionnaire. *Front. Psychol.* 7:650. 10.3389/fpsyg.2016.00650 27252663 PMC4879658

[B26] Chua A. Y. Goh D. H. L. Lee C. S. (2012). Mobile content contribution and retrieval: an exploratory study using the uses and gratifications paradigm. *Inform. Process. Manag.* 48 13–22. 10.1016/j.ipm.2011.04.002

[B27] Clifford K. J. Joyner K. H. Stroud D. B. Wood M. Ward B. Fernandez C. H. (1994). Mobile telephones interfere with medical electrical equipment. *Austr. Phys. Eng. Sci. Med.* 17 23–27. 8198505

[B28] Collins N. Read S. (1990). Adult attachment, working models, and relationship quality in dating couples. *J. Pers. Soc. Psychol.* 54 644–663. 10.1037/0022-3514.58.4.644 14570079

[B29] Contractor A. A. Weiss N. H. Tull M. T. Elhai J. D. (2017). PTSD’s relation with problematic smartphone use: mediating role of impulsivity. *Comput. Hum. Behav.* 75 177–183. 10.1016/j.chb.2017.05.019 PMC13099003 42023016

[B30] Csibi S. Demetrovics Z. Szabo A. (2016). Development and psychometric validation of the brief smartphone addiction scale (BSAS) with schoolchildren. *Psychiatr. Hungar.* 31 71–77. 27091924

[B31] Csibi S. Griffiths M. D. Cook B. Demetrovics Z. Szabo A. (2017). The psychometric properties of the smartphone application-based addiction scale (SABAS). *Intern. J. Ment. Health Add.* 16 393–403. 10.1007/s11469-017-9787-2 29670500 PMC5897481

[B32] David P. Kim J. Brickman J. S. Ran W. Curtis C. M. (2015). Mobile phone distraction while studying. *New Media Soc.* 17 1661–1679. 10.1177/1461444814531692

[B33] De-Sola J. Talledo H. Rubio G. de Fonseca F. R. (2017a). Development of a mobile phone addiction craving scale and its validation in a spanish adult population. *Front. Psychiatr.* 8:90. 10.3389/fpsyt.2017.00090 28611692 PMC5447711

[B34] De-Sola J. Talledo H. Rubio G. de Fonseca F. R. (2017b). Psychological factors and alcohol use in problematic mobile phone use in the Spanish population. *Front. Psychiatr.* 8:11. 10.3389/fpsyt.2017.00011 28217101 PMC5291168

[B35] DeVellis R. F. (2016). *Scale development: Theory and applications*, Vol. 26 Thousand Oaks, CA: Sage publications.

[B36] Ding J. Liu W. Wang X. Lan Y. Hu D. Xu Y. (2018). Development of a smartphone overuse classification scale. *Addict. Res. Theor.* 27 150–155. 10.1080/16066359.2018.1474204

[B37] Domoff S. E. Harrison K. Gearhardt A. N. Gentile D. A. Lumeng J. C. Miller A. L. (2019). Development and validation of the problematic media use measure: a parent report measure of screen media “addiction” in children. *Psychol. Popul. Media Cult.* 8 2–11. 10.1037/ppm0000163 30873299 PMC6411079

[B38] Ehrenberg A. Juckes S. White K. M. Walsh S. P. (2008). Personality and self-esteem as predictors of young people’s technology use. *Cyberpsychol. Behav.* 11 739–741. 10.1089/cpb.2008.0030 18991531

[B39] Eide T. A. Aarestad S. H. Andreassen C. S. Bilder R. M. Pallesen S. (2018). Smartphone restriction and its effect on subjective withdrawal related scores. *Front. Psychol.* 9:1444. 10.3389/fpsyg.2018.01444 30150959 PMC6099124

[B40] Elhai J. D. Dvorak R. D. Levine J. C. Hall B. J. (2017). Problematic smartphone use: a conceptual overview and systematic review of relations with anxiety and depression psychopathology. *J. Affect. Disord.* 207 251–259. 10.1016/j.jad.2016.08.030 27736736

[B41] Elhai J. D. Levine J. C. Alghraibeh A. M. Alafnan A. A. Aldraiweesh A. A. Hall B. J. (2018). Fear of missing out: testing relationships with negative affectivity, online social engagement, and problematic smartphone use. *Comput. Hum. Behav.* 89 289–298. 10.1016/j.chb.2018.08.020

[B42] Elhai J. D. Levine J. C. Dvorak R. D. Hall B. J. (2016). Fear of missing out, need for touch, anxiety and depression are related to problematic smartphone use. *Comput. Hum. Behav.* 63 509–516. 10.1016/j.chb.2016.05.079

[B43] Ellis R. (2017). *Look Left, Look Right – But Not At Your Cell Phone In Honolulu Crosswalks.* Available online at: http://www.cnn.com/2017/07/29/us/smart-phones-crosswalks-hawaii-illegal/index.html (accessed November 16, 2017).

[B44] Etter J.-F. (2005). A self-administered questionnaire to measure cigarette withdrawal symptoms: the cigarette withdrawal scale. *Nicot. Tobac. Res.* 7 47–57. 10.1080/14622200412331328501 15804677

[B45] Fidan H. (2016). Development and validation of the mobile addiction scale: the components model approach. *Addicta* 3 452–469. 10.15805/addicta.2016.3.0118

[B46] Flanagin A. J. (2005). IM online: instant messaging use among college students. *Commun. Res. Rep.* 3 175–187. 10.1080/00036810500206966

[B47] Foerster M. Roser K. Schoeni A. Röösli M. (2015). Problematic mobile phone use in adolescents: derivation of a short scale MPPUS-10. *Intern. J. Public Health* 60 277–286. 10.1007/s00038-015-0660-4 25645102

[B48] Google (2019). *Digital Wellbeing [Mobile application software].* Available online at: https://wellbeing.google/.

[B49] Grellhesl M. Punyanunt-Carter N. M. (2012). Using the uses and gratifications theory to understand gratifications sought through text messaging practices of male and female undergraduate students. *Comput. Hum. Behav.* 28 2175–2181. 10.1016/j.chb.2012.06.024

[B50] Griffiths M. (2005). A ‘components’ model of addiction within a biopsychosocial framework. *J. Substan. Use* 10 191–197. 10.1080/14659890500114359

[B51] Griffiths M. D. (2013). Adolescent mobile phone addiction: a cause for concern? *Educ. Health* 31 76–78.

[B52] Güzeller C. O. Coşguner T. (2012). Development of a problematic mobile phone use scale for Turkish adolescents. *Cyberpsychol. Behav. Soc. Netw.* 15 205–211. 10.1089/cyber.2011.0210 22304426

[B53] Ha J. Chin B. Park D. Ryu S. Yu J. (2008). Characteristics of excessive cellular phone use in Korean adolescents. *Cyberpsychol. Behav.* 11 783–784. 10.1089/cpb.2008.0096 18991536

[B54] Hadar A. Hadas I. Lazarovits A. Alyagon U. Eliraz D. Zangen A. (2017). Answering the missed call: initial exploration of cognitive and electrophysiological changes associated with smartphone use and abuse. *PLoS One* 12:e0180094. 10.1371/journal.pone.0180094 28678870 PMC5497985

[B55] Hawi N. S. Samaha M. (2016). To excel or not to excel: strong evidence on the adverse effect of smartphone addiction on academic performance. *Comput. Educ.* 98 81–89. 10.1016/j.compedu.2016.03.007

[B56] Hong F. Chiu S. S. Huang D. (2012). A model of the relationship between psychological characteristics, mobile phone addiction and use of mobile phones by Taiwanese university female students. *Comput. Hum. Behav.* 28 2152–2159. 10.1016/j.chb.2012.06.020

[B57] Honolulu, Hawaii, Ordinance 17-39, Bill 6 (2017). *City Council.* Hawaii: City and County of Honolulu.

[B58] Hooper V. Zhou Y. (2007). “Addictive, dependent, compulsive? A study of mobile phone usage,” in *Proceeding of the 20th Bled Conference Emergence: Merging and Emerging Technologies, Processes, and Institutions*, Bled.

[B59] Hope L. (2013). Help, we’re addicted to our smartphones. *Science* 21:17.

[B60] Hsieh Y.-P. Yen C.-F. Chou W.-J. (2019). Development and validation of the parental smartphone use management scale (PSUMS): parents’ perceived self-efficacy with adolescents with attention deficit hyperactivity disorder. *Intern. J. Environ. Res. Public Health* 16:1423. 10.3390/ijerph16081423 31010068 PMC6517877

[B61] Hunter S. C. Houghton S. Zadow C. Rosenberg M. Wood L. Shilton T. (2017). Development of the adolescent preoccupation with screens scale. *BMC Public Health* 17:652. 10.1186/s12889-017-4657-1 28800761 PMC5553924

[B62] Hussain Z. Griffiths M. D. Sheffield D. (2017). An investigation into problematic smartphone use: the role of narcissism, anxiety, and personality factors. *J. Behav. Add.* 6 378–386. 10.1556/2006.6.2017.052 28849667 PMC5700726

[B63] Igarashi T. Motoyoshi T. Takai J. Yoshida T. (2005). “The text messaging addiction scale: factor structure, reliability, and validity,” in *Paper presented at the sixth biennial conference of the Asian Association of Social Psychology*, Wellington.

[B64] Jenaro C. Flores N. Gómez-Vela M. González-Gil F. Caballo C. (2007). Problematic internet and cell-phone use: psychological, behavioral, and health correlates. *Add. Res. Theory* 15 309–320. 10.1080/16066350701350247

[B65] Kang H. Y. Park C. H. (2012). Development and validation of the smart phone addiction inventory. *Korea. J. Psychol.* 31 563–580.

[B66] Kaptsis D. King D. L. Delfabbro P. H. Gradisar M. (2016). Withdrawal symptoms in internet gaming disorder: a systematic review. *Clin. Psychol. Rev.* 43 58–66. 10.1016/j.cpr.2015.11.006 26704173

[B67] Karadağ E. Tosuntaş Ş.B. Erzen E. Duru P. Bostan N. Şahin B. M. (2015). Determinants of phubbing, which is the sum of many virtual addictions: a structural equation model. *J. Behav. Add.* 4 60–74. 10.1556/2006.4.2015.005 26014669 PMC4500886

[B68] Kim D. Lee Y. Lee J. Nam J. K. Chung Y. (2014). Development of Korean smartphone addiction proneness scale for youth. *Plos One* 9:e97920. 10.1371/journal.pone.0097920 24848006 PMC4029762

[B69] King A. L. S. Valença A. M. Silva A. C. Sancassiani F. Machado S. Nardi A. E. (2014). “Nomophobia”: impact of cell phone use interfering with symptoms and emotions of individuals with panic disorder compared with a control group. *Clin. Pract. Epidemiol. Ment. Health* 10:28. 10.2174/1745017901410010028 24669231 PMC3962983

[B70] Konok V. Gigler D. Bereczky B. M. Miklósi Á (2016). Humans’ attachment to their mobile phones and its relationship with interpersonal attachment style. *Comput. Hum. Behav.* 61 537–547. 10.1016/j.chb.2016.03.062

[B71] Koo H. Y. (2009). Development of a cell phone addiction scale for Korean adolescents. *J. Korea. Acad. Nurs.* 39 818–828. 10.4040/jkan.2009.39.6.818 20071895

[B72] Kumcagiz H. Gündüz Y. (2016). Relationship between psychological well-being and smartphone addiction of university students. *Intern. J. Higher Educ.* 5 144–156.

[B73] Kuss D. J. Harkin L. Kanjo E. Billieux J. (2018). Problematic smartphone use: Investigating contemporary experiences using a convergent design. *Intern. J. Environ. Res. Public Health* 15:142. 10.3390/ijerph15010142 29337883 PMC5800241

[B74] Kwon M. Kim D. Cho H. Yang S. (2013a). The smartphone addiction scale: development and validation of a short version for adolescents. *PLoS One* 8:e83558. 10.1371/journal.pone.0083558 24391787 PMC3877074

[B75] Kwon M. Lee J. Y. Won W. Y. Park J. W. Min J. A. Hahn C. (2013b). Development and validation of a smartphone addiction scale (SAS). *PLoS One* 8:e56936. 10.1371/journal.pone.0056936 23468893 PMC3584150

[B76] Labrador Encinas J. Villadangos González S. M. (2010). Menores y nuevas tecnologías: Conductas indicadoras de posible problema de adicción. *Psicothema* 22 180–188. 10.20882/adicciones.107 20423619

[B77] Laramie D. J. (2007). Emotional and behavioral aspects of mobile phone use. *Dissert. Abstr. Intern.* 68:4136.

[B78] Lee H. Kim J. Fava M. Mischoulon D. Park J. Shim E. (2017). Development and validation study of the smartphone overuse screening questionnaire. *Psychiatr. Res.* 257 352–357. 10.1016/j.psychres.2017.07.074 28800515

[B79] Leung L. (2008). Linking psychological attributes to addiction and improper use of the mobile phone among adolescents in Hong Kong. *J. Child. Media* 2 93–113. 10.1080/17482790802078565

[B80] Lin Y. Pan Y. Lin S. Chen S. (2017). Development of short-form and screening cutoff point of the smartphone addiction inventory (SPAI-SF). *Intern. J. Methods Psychiatr. Res.* 26:e001525. 10.1002/mpr.1525 27658956 PMC6877212

[B81] Lin Y. H. Chang L. R. Lee Y. H. Tseng H. W. Kuo T. B. Chen S. H. (2014). Development and validation of the smartphone addiction inventory (SPAI). *PLoS One* 9:e98312. 10.1371/journal.pone.0098312 24896252 PMC4045675

[B82] Lin Y. H. Chiang C. L. Lin P. H. Chang L. R. Ko C. H. Lee Y. H. (2016). Proposed diagnostic criteria for smartphone addiction. *PLoS One* 11:e0163010. 10.1371/journal.pone.0163010 27846211 PMC5112893

[B83] Lopez-Fernandez O. Kuss D. J. Pontes H. M. Griffiths M. D. Dawes C. Justice L. V. (2018). Measurement invariance of the short version of the problematic mobile phone use questionnaire (PMPUQ-SV) across eight languages. *Intern. J. Environ. Res. Public Health* 15:1213. 10.3390/ijerph15061213 29890709 PMC6025621

[B84] Martinotti G. Villella C. C. Di Thiene D. Di Nicola M. Bria P. Conte G. (2011). Problematic mobile phone use in adolescence: a cross-sectional study. *J. Public Health* 19 545–551. 10.1007/s10389-011-0422-6

[B85] Matar Boumosleh J. Jaalouk D. (2017). Depression, anxiety, and smartphone addiction in university students- a cross sectional study. *PLoS One* 12:0182239. 10.1371/journal.pone.0182239 28777828 PMC5544206

[B86] Merlo L. J. Stone A. M. Bibbey A. (2013). Measuring problematic mobile phone use: development and preliminary psychometric properties of the PUMP scale. *J. Add.* 2013:7. 10.1155/2013/912807 PMC4008508 24826371

[B87] Mokkink L. B. Terwee C. B. Patrick D. L. Alonso J. Stratford P. W. Knol D. L. (2013). *The COSMIN Checklist Manual.* Available online at: www.cosmin.nl (accessed August 2018).

[B88] Monge Roffarello A. De Russis L. (2019). “The race towards digital wellbeing: issues and opportunities,” in *Proceedings of the 2019 CHI Conference on Human Factors in Computing Systems*, (New York, NY: ACM), 386.

[B89] Nunnally J. Bernstein L. (1994). *Psychometric Theory.* New York, NY: McGraw-Hill Higher, INC.

[B90] O’Connor S. S. Shain L. M. Whitehill J. M. Ebel B. E. (2017). Measuring a conceptual model of the relationship between compulsive cell phone use, in-vehicle cell phone use, and motor vehicle crash. *Accid. Analy. Prevent.* 99(Pt A), 372–378. 10.1016/j.aap.2016.12.016 28068624

[B91] O’Connor S. S. Whitehill J. M. King K. M. Kernic M. A. Boyle L. N. Bresnahan B. W. (2013). Compulsive cell phone use and history of motor vehicle crash. *J. Adolesc. Health* 53 512–519. 10.1016/j.jadohealth.2013.05.015 23910571 PMC3786686

[B92] Olivencia-Carrion M. A. Ferri-Garcia R. Rueda M. Lopez-Torrecillas F. (2018a). Reliability and construct validity of a questionnaire to assess the nomophobic (QANIP). *BMC Public Health*

[B93] Olivencia-Carrion M. A. Ramirez-Ucles I. Holgado-Tello P. Lopez-Torrecillas F. (2018b). Validation of a spanish questionnaire on mobile phone abuse. *Front. Psychol.* 9:621. 10.3389/fpsyg.2018.00621 29760674 PMC5936979

[B94] Olufadi Y. (2015). Gravitating towards mobile phone (GoToMP) during lecture periods by students: why are they using it? And how can it be measured? *Comput. Educ.* 87 423–436. 10.1016/j.compedu.2015.08.013

[B95] Pamuk M. Atli A. (2016). Development of a problematic mobile phone use scale for university students: validity and reliability study. *Dusunen. Adam.* 29 49–59. 10.5350/DAJPN2016290105

[B96] Pancani L. Preti E. Riva P. (2019). The psychology of smartphone: the development of the smartphone impact scale (SIS). *Assessment* 10.1177/1073191119831788 [Epub ahead of print]. 30829048

[B97] Panova T. Carbonell X. (2018). Is smartphone addiction really an addiction? *J. Behav. Add.* 7 252–259. 10.1556/2006.7.2018.49 29895183 PMC6174603

[B98] Parasuraman S. Sam A. T. Yee S. Chuon B. Ren L. Y. (2017). Smartphone usage and increased risk of mobile phone addiction: a concurrent study. *Int. J. Pharm. Investig.* 7 125–131. 10.4103/jphi.JPHI_56_17 29184824 PMC5680647

[B99] Pawłowska B. Potembska E. (2009). Właściwości psychometryczne kwestionariusza do badania uzależnienia od telefonu komórkowego (KBUTK). *Badania Schizofrenia?* 10 322–329.

[B100] Pedrero-Pérez E. J. Rodríguez-Monje M. T. Gallardo-Alonso F. Fernández-Girón M. Pérez-López M. Chicharro-Romero J. (2007). Validación de un instrumento para la detección de trastornos de control de impulsos y adicciones: el MULTICAGE CAD-4. *Trastorn. Adict.* 9 269–278. 10.1016/S1575-0973(07)75656-75658

[B101] Pedrero-Pérez E. J. Ruiz Sánchez de León J. M. Rojo Mota G. Llanero Luque M. Pedrero Aguilar J. Morales Alonso S. (2018). Information and Communications Technologies (ICT): problematic use of Internet, video games, mobile phones, instant messaging and social networks using MULTICAGE-TIC. *Adicciones* 30 19–32. 10.20882/adicciones.806 28492951

[B102] Pew Research Center (2018). *Smartphone Ownership in Advanced Economies Higher Than In Emerging [Infographic].* Available online at: https://www.pewresearch.org/global/2019/02/05/smartphone-ownership-is-growing-rapidly-around-the-world-but-not-always-equally/pg_global-technology-use-2018_2019-02-05_0-01/ (accessed May 11, 2018).

[B103] Potenza M. N. (2014). Non-substance addictive behaviors in the context of DSM-5. *Add. Behav.* 39 1–2. 10.1016/j.addbeh.2013.09.004 24119712 PMC3858502

[B104] Potenza M. N. Higuchi S. Brand M. (2018). Call for research into a wider range of behavioural addictions. *Nature* 555:7694. 10.1038/d41586-018-02568-z 29493605

[B105] Roberts J. A. David M. E. (2016). My life has become a major distraction from my cell phone: partner phubbing and relationship satisfaction among romantic partners. *Comput. Hum. Behav.* 54 134–141. 10.1016/j.chb.2015.07.058

[B106] Roberts J. A. Pullig C. Manolis C. (2015). I need my smartphone: a hierarchical model of personality and cell-phone addiction. *Pers. Individ. Differ.* 79 13–19. 10.1016/j.paid.2015.01.049

[B107] Roberts J. A. YaYa L. H. P. Manolis C. (2014). The invisible addiction: cell-phone activities and addiction among male and female college students. *J. Behav. Add.* 3 254–265. 10.1556/jba.3.2014.015 25595966 PMC4291831

[B108] Rosen L. D. Whaling K. Carrier L. M. Cheever N. A. Rokkum J. (2013). The media and technology usage and attitudes scale: an empirical investigation. *Comput. Hum. Behav.* 29 2501–2511. 10.1016/j.chb.2013.06.006 25722534 PMC4338964

[B109] Rozgonjuk D. Rosenvald B. Janno S. Täht K. (2016). Developing a shorter version of the estonian smartphone addiction proneness scale (E-SAPS18). *Cyberpsychol. J. Psychosoc. Res. Cyberspace* 10 4 10.5817/CP2016-4-4

[B110] Ruggiero T. E. (2000). Uses and gratifications theory in the 21st century. *Mass Commun. Soc.* 3 3–37. 10.1207/s15327825mcs0301_02

[B111] Rutland J. B. Sheets T. Young T. (2007). Development of a scale to measure problem use of short message service: the SMS problem use diagnostic questionnaire. *Cyberpsychol. Behav.* 10 841–843. 10.1089/cpb.2007.9943 18085975

[B112] Samaha M. Hawi N. S. (2016). Relationships among smartphone addiction, stress, academic performance, and satisfaction with life. *Comput. Hum. Behav.* 57 321–325. 10.1016/j.chb.2015.12.045

[B113] Scott D. A. Valley B. Simecka B. A. (2016). Mental health concerns in the digital age. *Intern. J. Men. Health Add.* 15 604–613. 10.1007/s11469-016-9684-0

[B114] Sheth J. N. Newman B. I. Gross B. L. (1991). Why we buy what we buy: a theory of consumption values. *J. Bus. Res.* 22 159–170. 10.1016/0148-2963(91)90050-8

[B115] Shin L. Y. (2014). A comparative study of mobile internet usage between the U.S. and Korea. *J. Eur. Psychol. Stud.* 5 46–55. 10.5334/jeps.cg

[B116] Smetaniuk P. (2014). A preliminary investigation into the prevalence and prediction of problematic cell phone use. *J. Behav. Add.* 3 41–53. 10.1556/JBA.3.2014.004 25215213 PMC4117273

[B117] Starcevic V. (2013). Is internet addiction a useful concept? *Aust. N Z J. Psychiatry* 47 16–19. 10.1177/0004867412461693 23293309

[B118] Starcevic V. (2016). Tolerance and withdrawal symptoms may not be helpful to enhance understanding of behavioural addictions. *Addiction* 111 1307–1308. 10.1111/add.13381 27095413

[B119] Su S. Pan T. T. Liu X. Q. Chen X. W. Wang Y. J. Li M. Y. (2014). Development of the smartphone addiction scale for college students. *Chin. Ment. Health J.* 28 392–397.

[B120] Tao S. M. Fu J. L. Wang H. Hao J. H. Tao F. B. (2013). Development of self-rating questionnaire for adolescent problematic mobile phone use and the psychometric evaluation in undergraduates. *Chin. J. Sch. Health* 34 26–29.

[B121] Thompson B. (ed.) (2003). *Score Reliability: Contemporary Thinking On Reliability Issues.* Thousand Oaks, CA: Sage Publications Inc.

[B122] Toda M. Monden K. Kubo K. Morimoto K. (2004). Cellular phone dependence tendency of female university students. *Jpn. J. Hyg.* 59 383–386. 10.1265/jjh.59.383 15626025

[B123] Tossell C. Kortum P. Shepard C. Rahmati A. Zhong L. (2015). Exploring smartphone addiction: insights from long-term telemetric behavioral measures. *Intern. J. Inter. Mobile Technol.* 9 37–43. 10.3991/ijim.v9i2.4300

[B124] Trub L. Barbot B. (2016). The paradox of phone attachment: development and validation of the young adult attachment to phone scale (YAPS). *Comput. Hum. Behav.* 64 663–672. 10.1016/j.chb.2016.07.050

[B125] Twice Staff (2018). *Smartphones Could Reach TV Ownership Rates Within 5 Years.* Available online at: https://www.twice.com/product/smartphones-could-reach-tv-ownership-rates-within-5-years-cta (accessed July 26, 2018).

[B126] Valderrama J. A. (2014). *Development and Validation of the Problematic Smartphone Use Scale.* Doctoral dissertation (Alhambra, CA: Alliant International University).

[B127] van Deursen A. J. A. M. Bolle C. L. Hegner S. M. Kommers P. A. M. (2015). Modeling habitual and addictive smartphone behavior: the role of smartphone usage types, emotional intelligence, social stress, self-regulation, age, and gender. *Comput. Hum. Behav.* 45 411–420. 10.1016/j.chb.2014.12.039

[B128] Walsh S. P. White K. M. McD Young R. (2010). Needing to connect: the effect of self and others on young people’s involvement with their mobile phones. *Austr. J. Psychol.* 62 194–203. 10.1080/00049530903567229

[B129] Weed N. Butcher N. J. McKenna T. Ben-Porath Y. (1992). New measures for assessing alcohol and other drug problems with the MMPI-2: The APS and AAS. *J. Pers. Assess.* 58 389–404. 10.1207/s15327752jpa5802_15 1315859

[B130] World Health Organization [WHO] (2018). *International Statistical Classification Of Diseases and Related Health Problems (11th Revision).* Geneva: WHO.

[B131] Xiong J. Zhou Z. Chen W. You Z. Zhai Z. (2012). Development of the mobile phone addiction tendency scale for college students. *Chine. Ment. Health J.* 26 222–225.

[B132] Yen C. Tang T. Yen J. Lin H. Huang C. Liu S. (2009). Symptoms of problematic cellular phone use, functional impairment and its association with depression among adolescents in Southern Taiwan. *J. Adolesc.* 32 863–873. 10.1016/j.adolescence.2008.10.006 19027941

[B133] Yildirim C. Correia A. (2015). Exploring the dimensions of nomophobia: development and validation of a self-reported questionnaire. *Comput. Hum. Behav.* 49 130–137. 10.1016/j.chb.2015.02.059

[B134] Young K. S. (1996). Psychology of computer use: XL. Addictive use of the internet: a case that breaks the stereotype. *Psychol. Rep.* 79(Pt. 1), 899–902. 10.2466/pr0.1996.79.3.899 8969098

[B135] Young K. S. (1998). Internet addiction: the emergence of a new clinical disorder. *Cyberpsychol. Behav.* 1 237–244. 10.1089/cpb.1998.1.237

